# Evaluation of the safety and efficacy of *Sophorae Flavescentis* Radix extract in the treatment of inflammatory bowel disease based on zebrafish models

**DOI:** 10.3389/fimmu.2025.1722777

**Published:** 2025-11-27

**Authors:** Xiaolu Chen, Muyao Cui, Jiaqi Li, Qi Chen, Linzhen Chen, Zhuo Yang, Qiqi Fan, Yuhan Sheng, Ruichao Lin, Zhiqiang Ma, Chongjun Zhao

**Affiliations:** 1 Beijing Key Laboratory for Quality Evaluation of Chinese Materia Medica, School of Chinese Materia Medica, Beijing University of Chinese Medicine, Beijing, China; 2 Beijing University of Chinese Medicine, Beijing, China; 3 College of Traditional Chinese Medicine, Beijing University of Chinese Medicine, Beijing, China

**Keywords:** *Sophorae Flavescentis* Radix, IBD, zebrafish, safety, effectiveness, mechanism

## Abstract

**Background:**

*Sophorae Flavescentis* Radix (Kushen) has been employed in Traditional Chinese Medicine (TCM) for over 2,000 years, primarily for its effects in clearing heat and drying dampness, eliminating parasites, and promoting diuresis. Preliminary findings suggest that Kushen extract or its formulations may exhibit potential therapeutic benefits in alleviating inflammatory bowel disease (IBD).

**Aims:**

This study aimed to comprehensively evaluate the safety and efficacy of Kushen extract using a zebrafish model, with a particular focus on intestinal mucosal immunity, and to explore its underlying mechanisms.

**Material and methods:**

The safety and efficacy profile of Kushen extract was comprehensively evaluated in zebrafish model. Network pharmacology combined with transcriptomics were employed to predict the mechanisms underlying Kushen extract’s therapeutic effects on IBD, followed by validation using Reverse Transcription-Quantitative Polymerase Chain Reaction (RT-qPCR) and molecular docking.

**Results:**

Administration of supra-therapeutic doses of Kushen extract induced hepatotoxicity effects in zebrafish, primarily manifested as hepatic morphological abnormalities and exacerbated hepatocyte apoptosis. Conversely, low-dose administration of Kushen extract demonstrated significant therapeutic benefits including improved intestinal phagocytic function, reduced gut neutrophil infiltration and enhanced goblet cell secretion. Furthermore, Kushen extract treatment at low doses significantly decreased the levels of pro-inflammatory cytokines. Integrated network pharmacology and transcriptomic analyses indicated that the amelioration of Kushen extract on IBD may be involved in FoxO4/NOD-like/Apoptosis and MAPK signaling pathways. The RT-qPCR results confirmed that Kushen extract effectively modulates the expression levels of key genes in the aforementioned pathway. Molecular docking analysis revealed binding energies of -8.1 kcal/mol between matrine and the BCL-2 protein, and -10.4 kcal/mol between oxymatrine and EGFR, indicating strong molecular interactions between these compounds and their target proteins. These interactions may collectively contribute to the therapeutic efficacy of Kushen extract.

**Conclusions:**

Supra-therapeutic doses of Kushen extract can induce marked liver damage in zebrafish. However, when administration within an appropriate dosage range, it does not elicit significant toxicity and demonstrates substantial therapeutic efficacy against IBD, partly through modulating mucosal immunity. These findings provide crucial experimental evidence to support the guidance for safe and rational clinical application of Kushen extract.

## 1 Introduction

Inflammatory bowel disease (IBD) is a complex disorder characterized by chronic inflammation of the gastrointestinal tract, primarily encompassing Crohn’s disease (CD) and ulcerative colitis (UC). The pathogenesis remains incompletely elucidated, while it is widely acknowledged to be closely associated with environmental factors, infectious microorganisms, ethnicity, genetic predisposition, and immune system dysregulation (1–4). Of particular concern is the persistent rise in incidence globally (5, 6), which has increasingly evolved into a substantial burden on public health. Furthermore, a considerable proportion of patients exhibit inadequate responses to current therapies (7). Consequently, there is an urgent clinical need to develop novel IBD therapeutic agents or strategies that are both more effective and possess a favorable safety profile.

Against this backdrop, the Chinese herbal medicine *Sophorae Flavescentis* Radix (Kushen) has garnered considerable research interest. This interest stems from its long history of medicinal use, documented efficacy in traditional applications such as clearing heat and drying dampness (8), antiparasitic activity (9), and diuretic effects (10, 11), as long as modern pharmacological studies confirming its anti-inflammatory, anti-tumor, and analgesic properties (12–14). Notably, preliminary findings from several clinical studies suggest that Kushen extract or its formulations demonstrate potential therapeutic efficacy in alleviating symptoms in patients suffered with IBD (15–18). This observed modern efficacy corresponds with its documented 2,000-year history of use in treating intestinal inflammation, as recorded in the *Shennong Ben Cao Jing* (ca. 100 CE) (19), which explicitly describes its capacity to “clear heat, dry dampness, and relieve dysentery”. However, these studies also indicate that the precise mechanisms underlying Kushen extract’s therapeutic effects remain poorly understood and warrant further investigation. The primary active constituents of Kushen extract include alkaloids (e.g., matrine, sophoridine, oxymatrine) and flavonoids. Among these, matrine is considered a major active component, however, accumulating evidence suggest it may induce hepatotoxicity (20, 21), leading to adverse events and imposing limitations on its clinical safety. More critically, there is currently a lack of systematic and in-depth understanding of Kushen extract’s toxicity-efficacy relationship. This ambiguity surrounding the relationship not only poses multifaceted challenges for clinical practice and patient safety, but also hinders the development of safer and more effective derivatives from Kushen. Therefore, investigating the Kushen extract’s toxicity-efficacy relationship and its underlying mechanisms not only possesses significant scientific importance but also offers profound clinical value. Such research is essential for guiding the safe and rational clinical use, mitigating potential risks, and facilitating the development of safer and more effective drugs derived from Kushen extract. It constitutes an indispensable pathway towards the modernization and international application of this traditional medicine herb.

The zebrafish (*Danio rerio*) has emerged as a well-established vertebrate model organism in biomedical research (22, 23). With approximately 70% genomic homology to humans (24), this model demonstrates a broader range of disease-relevant phenotypes than invertebrate models and exhibits features conducive to high-throughput screening (25). Consequently, it is extensively employed in drug safety evaluation and has become a vital model for investigating disease mechanisms and conducting drug discovery studies (26–28). Network pharmacology offers a systematic approach to constructing interaction networks among “herbal components - targets - diseases”, providing novel insights into elucidating the material basis and their mechanisms of action of traditional Chinese medicines in treating diseases (29, 30). Transcriptomics technology enables the precise identification of differentially expressed genes (DEGs) and their associated signaling pathways, as well as the screening of potential biomarkers and novel drug targets (31, 32). Therefore, the integration of network pharmacology and transcriptomics facilitates a multi-dimensional and multi-level analysis of the pharmacological mechanisms underlying herbal medicines.

**Figure 1:**
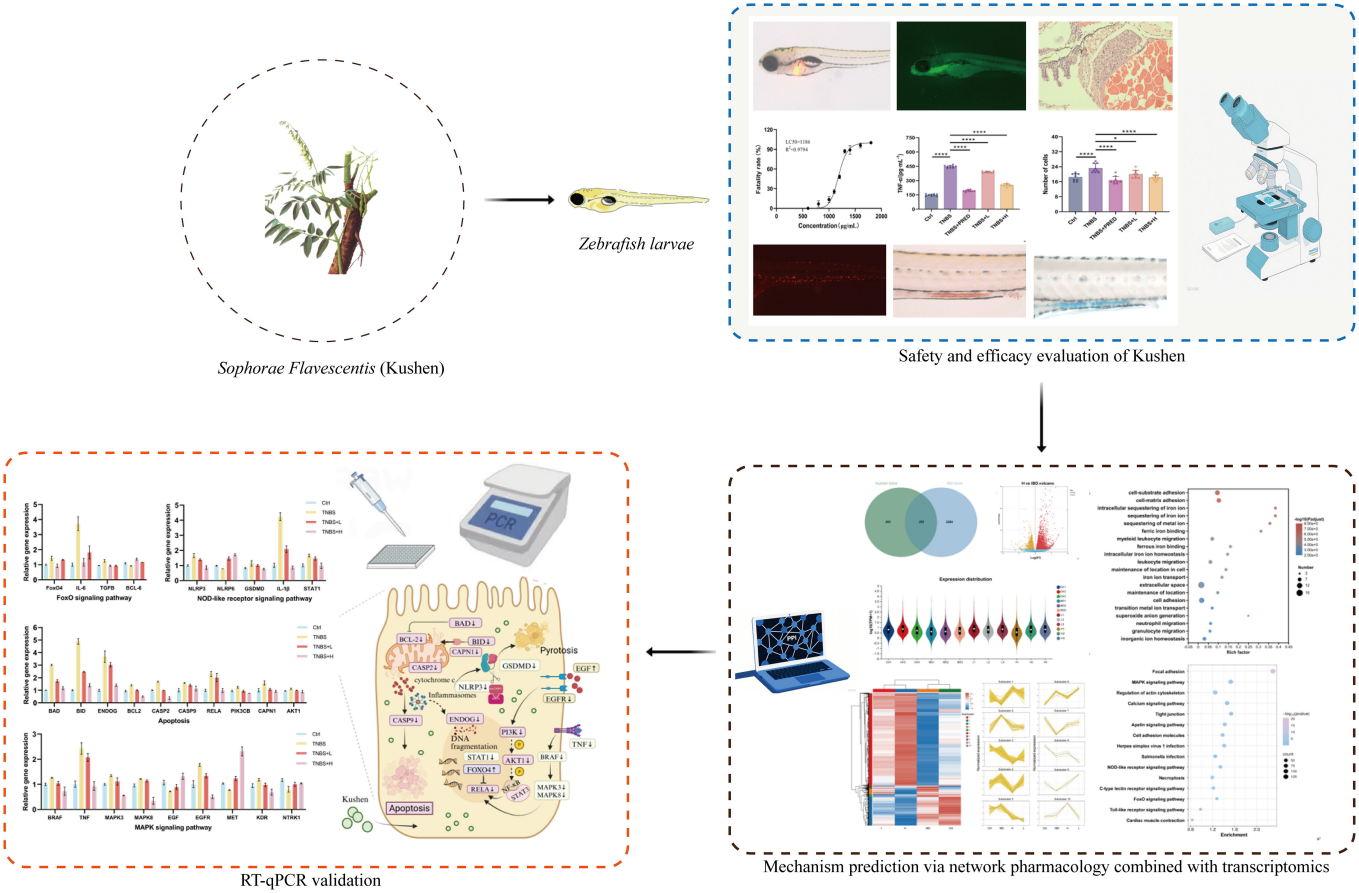
Graphical abstract.

This study initially evaluated the safety profile of Kushen extract using the zebrafish model. Subsequently, a zebrafish model of IBD was established using TNBS induction to assess the therapeutic efficacy of Kushen extract. Furthermore, an integrated approach combining network pharmacology and transcriptomics was employed to predict the mechanisms underlying Kushen extract’s effects on IBD, leading to the identification of key DEGs and associated pathways. Finally, these predictions were subsequently validated using real-time quantitative PCR (RT-qPCR). Collectively, this work aims to provide a scientific foundation for the safe clinical application of Kushen extract and the treatment of IBD (**Figure 1**).

## 2 Materials and methods

### 2.1 Zebrafish breeding and handling

Wild-type AB strain zebrafish and transgenic lines CZ16 (Tg(ela3l: EGFP)) and CZ59 (Tg(lyz: DsRED2)) used in this study were maintained within the zebrafish housing system of the Beijing Key Laboratory for Quality Evaluation of Traditional Chinese Medicine. The water temperature was controlled at 28.0 ± 0.5°C, with the pH level maintained within the range of 7.0 to 7.4. A photoperiod of 14 hours of light and 10 hours of darkness was applied. Fertilized eggs were obtained from 6-month-old adult zebrafish during spawning and incubated in zebrafish culture water at 28.5 °C until 4 days post-fertilization (4 dpf). Both the incubation process and subsequent experiments procedures were conducted in a controlled incubator with constant temperature and humidity. All animal handling procedures and experimental conditions were approved by the Animal Ethics Committee of Beijing University of Chinese Medicine, China.

### 2.2 Reagents and instruments

Crude kushen sample (Batch No.: 2308000098) were purchased from Anguo Anxing Co., Ltd. The identity and quality of the Kushen were verified by the supplier in accordance with the specifications outlined in the Chinese Pharmacopoeia (2020 edition). Prednisolone (PRED, Batch No. L2205524, Aladdin). 2,4,6-trinitrobenzenesulfonic acid (TNBS; Batch No. MB5523, Dalian Meilun Biotechnology Co., Ltd.). Acridine orange (AO; Batch No.: 240104001, Solarbio). Methyl cellulose (Batch No. A15GS145385, Yuanye Bio-Technology Co., Ltd.). Hematoxylin and Eosin (H&E) staining kit (Batch No. B1003, Wuhan Baqiandu Biotechnology Co., Ltd.). 4% Paraformaldehyde (PFA; Batch No. 24018549, Biosharp). Glacial acetic acid (Batch No. 20191005, Damiao Chemical Reagent Factory). Alcian Blue (Batch No. SL30173318, Coolaber). Neutral Red (Batch No. 20210114, Solarbio). Enzyme-linked immunosorbent assay (ELISA) kits for Interleukin-1 beta (IL-1β; Batch No. MK0036FA), Tumor Necrosis Factor-alpha (TNF-α; Batch No. MK530049A), and Prostaglandin E2 (PGE2; Batch No. MK530265A) were purchased from Jiangsu Sumaike Biotechnology Co., Ltd. TRIzol reagent (Batch No. 1000092821, Invitrogen/Thermo Fisher Scientific). Reverse Transcription Kit (Batch No. F0202-100T, LabEAD). First Strand cDNA Synthesis Kit (Batch No. 0202120731, LabEAD). Zebrafish recirculating aquaculture system (Model: ESEN-AW-S1, Aisheng Biotechnology Co., Ltd.). Stereomicroscope with fluorescence capability (Model: Zeiss Axio Zoom. V16, Carl Zeiss AG). Ultraviolet-Visible microvolume spectrophotometer (Model: NanoDrop 2000, Thermo Fisher Scientific). Real-Time PCR System (Model: StepOnePlus, Thermo Fisher Scientific).

### 2.3 Preparation of Kushen extract sample

The Kushen extract used in this study was prepared by our research team from the purchased crude drug. An appropriate amount of Kushen sample was immersed in six times its volume of acetic acid solution. Percolation was subsequently conducted with two volumes of acidified water and the resulting percolate was collected. Subsequently, the residual marc was decocted with water, and the obtained decoction was retained. The combined liquid extracts were concentrated under reduced pressure, dried, and ground into a fine powder to obtain the Kushen extract. The chemical profile of the Kushen extract was further confirmed to meet the pharmacopoeial requirements through HPLC analysis (in **Supplementary Figures**). The extract was then dissolved in zebrafish water to the desired concentration, yielding the final Kushen extract solution for use in subsequent experiments.

### 2.4 Safety evaluation of Kushen extract

Healthy CZ16 transgenic zebrafish (4 dpf) were randomly allocated to individual 12-well plates containing test solutions of Kushen extract at various concentrations, with 20 zebrafish per well. Concurrently, a control group was simultaneously established using zebrafish culture water. The entire exposure process was conducted under controlled conditions at a constant temperature of 28 °C. After 24 hours of exposure, the number of deceased zebrafish in each group was recorded, and a dose-toxicity curve was constructed to determine the 10% lethal concentration (LC_10_) and the maximum non-lethal concentration (LC_0_). Subsequently, two distinct dosing regimens were selected within the sub-lethal concentration range (< LC_10_). According to the experimental design, specific methodologies were employed to comprehensively assess the effects of Kushen extract on zebrafish liver function from multiple perspectives. At the conclusion of the experiment, the exposure solution was replaced with a 5 μg·mL^−1^ AO solution and incubated for 30 minutes in the dark. Subsequently, apoptosis of liver cells in zebrafish from different groups was evaluated under a fluorescence microscope. Additionally, the lateral position of CZ16 transgenic zebrafish, which had been anesthetized with tricaine, was fixed on a slide coated with sodium methylcellulose for observation and photography. The liver fluorescence area and optical density were analyzed quantitatively using Image-Pro Plus software. Finally, zebrafish were transferred to 4% PFA for fixation, followed by sequential steps of dehydration, paraffin embedding, sectioning, and hematoxylin-eosin (HE) staining. The histological characteristics of zebrafish liver tissues in each group were systematically examined under a light microscope.

### 2.5 Evaluation of the therapeutic efficacy of Kushen extract in IBD

According to Xu et al. (33), 4dpf zebrafish were exposed to a 70 mg·L^−1^ TNBS solution for 24 hours under adjusted experimental conditions to establish an IBD model. Immediately following TNBS exposure, the experimental group of zebrafish was simultaneously administered a 24-hour treatment with Kushen extract at safe doses (lower than LC_0_: low dose 300 μg·mL^−1^ and high dose 500 μg·mL^−1^ ). The model control group was maintained in standard culture water without any intervention. Additionally, a positive control group was established by administering 30 mg·L^−1^ prednisolone, while the control group consisted of untreated zebrafish maintained in zebrafish culture water. At the conclusion of the experiment, appropriate analyses were performed in accordance with defined research objectives.

Specifically, following the anesthesia of CZ 59 neutrophil transgenic zebrafish, the number of neutrophils in the intestinal tract was quantified for each treatment group using a microscope and Image Pro Plus software. Additionally, neutral red staining solution was applied to all group samples, which were subsequently incubated in the dark at a constant temperature for 1 hour. After cleaning and re-anesthetizing the samples, the intestinal staining of zebrafish was observed and photographed under a microscope, with the stained area quantified using Image Pro Plus software. Furthermore, zebrafish from different groups were fixed in 4% PFA and stored overnight at 4 °C. The following day, after rinsing the samples with 1% hydrochloric acid ethanol, alcian blue staining solution was used for dark staining in a constant temperature incubator for 2 hours. Subsequent to another rinse with 1% hydrochloric acid ethanol, the intestinal staining area was analyzed and quantified using a microscope and Image Pro Plus software. For the ELISA assays, a total of 80 zebrafish larvae per experimental group were pooled to constitute one biological replicate, as sample pooling was necessary to obtain an adequate volume of homogenate for reliable quantification of cytokine levels. The concentrations of IL-1β, TNF-α, and PGE2 were measured in strict accordance with the manufacturer’s instructions. All experiments designated for statistical analysis were independently repeated three times at least to ensure data reproducibility and reliability.

### 2.6 Investigation of the mechanism of Kushen extract in treating IBD via network pharmacology and transcriptomics

#### 2.6.1 Network pharmacology analysis

A comprehensive compilation of the chemical constituents of Sophora flavescens was achieved by integrating information from the existing literature and the PubChem database (https://pubchem.ncbi.nlm.nih.gov/). The corresponding Simplified Molecular Input Line Entry System (SMILES) strings were subsequently input into the Swiss Target Prediction platform for predicting potential molecular targets. A search for the keyword “IBD” was conducted in the GeneCards database, and targets with scores exceeding the median value were selected. Thereafter, the predicted targets of Sophora flavescens were cross-referenced with established targets associated with IBD, and their intersection was identified as the potential therapeutic targets of Sophora flavescens in this context. Protein-protein interaction (PPI) analysis was performed using the STRING database (https://string-db.org/), and the resultant PPI network was visualized using Cytoscape 3.7.2 software. Furthermore, gene ontology (GO) functional enrichment analysis and Kyoto Encyclopedia of Genes and Genomes (KEGG) pathway enrichment analysis were executed via the Metascape database (https://metascape.org/). Finally, data visualization was accomplished using the Microbio informatics platform (http://www.bioinformatics.com.cn/login/).

#### 2.6.2 Transcriptomics analysis

Zebrafish at 4 dpf were exposed to a 70 mg·L^−1^ solution of TNBS to induce IBD. After successful model establishment, the experimental groups were randomly assigned to receive a 24-hour treatment with Kushen extract at concentrations of 300 μg·mL^−1^ and 500 μg·mL^−1^ , respectively. The model control group was maintained in standard culture water without any intervention. At the experimental endpoint, the drug solution was removed, and zebrafish were rinsed. A total of 120 zebrafish samples were collected per group. Total RNA was extracted from zebrafish using TRIzol reagent. The concentration and purity of the extracted RNA were assessed using a NanoDrop 2000 spectrophotometer, and RNA integrity was evaluated via agarose gel electrophoresis. mRNA was enriched using the Oligo dT method for transcriptome analysis. Fragmentation buffer was added to randomly fragment the mRNA. First-strand cDNA was synthesized using reverse transcriptase, followed by adapter ligation. Library construction was performed using the Illumina^®^ Stranded mRNA Prep, Ligation kit. Sequencing was carried out on the NovaSeq X Plus platform. Gene and transcript expression levels were quantified using RSEM software. DEGs were screened using DESeq2 with the threshold set at False Discovery Rate (FDR) < 0.05 and |log2 fold change (log2FC)| ≥ 1. Finally, GO and KEGG pathway enrichment analyses were conducted to annotate and interpret the biological information.

#### 2.6.3 Integrated network pharmacology and transcriptomics analysis

The potential targets identified through network pharmacology analysis were cross-referenced with the DEGs obtained from transcriptomics analysis to identify key targets involved in Kushen extract’s therapeutic effect on IBD. Bioinformatics platforms were utilized to perform correlation analysis on the intersected targets, followed by GO and KEGG enrichment analyses. Data visualization was performed using the MicrobioMed platform.

### 2.7 Validation of relevant gene expression by RT-qPCR

At the experimental endpoint, the drug solution was removed and zebrafish were rinsed. A total of 80 zebrafish samples were collected from each group. Total RNA was extracted using TRIzol reagent and subsequently reverse transcribed into cDNA using a Reverse Transcription Kit. Quantitative real-time PCR reactions were performed using a PCR kit on a StepOne Plus™ System (Applied Biosystems). The β-actin gene was used as the internal reference gene, and gene-specific primers were synthesized by Ruibo Xingke Biotechnology Co., Ltd. The relative expression levels of target genes were calculated using the comparative 2^-ΔΔCt^ method. The primers are listed in **Supplementary Table 1**.

### 2.8 Molecular docking

To investigate the interactions between the bioactive components of Kushen extract and core targets, this study focused on homologous targets conserved in zebrafish and humans, which were identified through integrated network pharmacology, transcriptomics, and RT-qPCR analyses, followed by molecular docking to assess binding affinity. The three-dimensional structures of key target proteins were retrieved from the Protein Data Bank (PDB; https://www.rcsb.org/), while the structural data of small-molecule compounds were obtained from the PubChem database. Both target proteins and ligands were prepared using AutoDock Tools 1.5.6. Molecular docking simulations were subsequently carried out using AutoDock Vina, and favorable binding conformations between proteins and ligands were visualized using PyMOL.

### 2.9 Statistical analysis

All experiments were performed in triplicate. Data were analyzed using GraphPad Prism software (version 9.5) and are presented as mean ± standard deviation (x ± s). Differences between groups were assessed by one-way analysis of variance (ANOVA) and T-test, with statistical significance defined as *p < 0.05 and high statistical significance defined as **p < 0.01.

## 3 Results

### 3.1 Safety evaluation of Kushen extract

**Figure 2:**
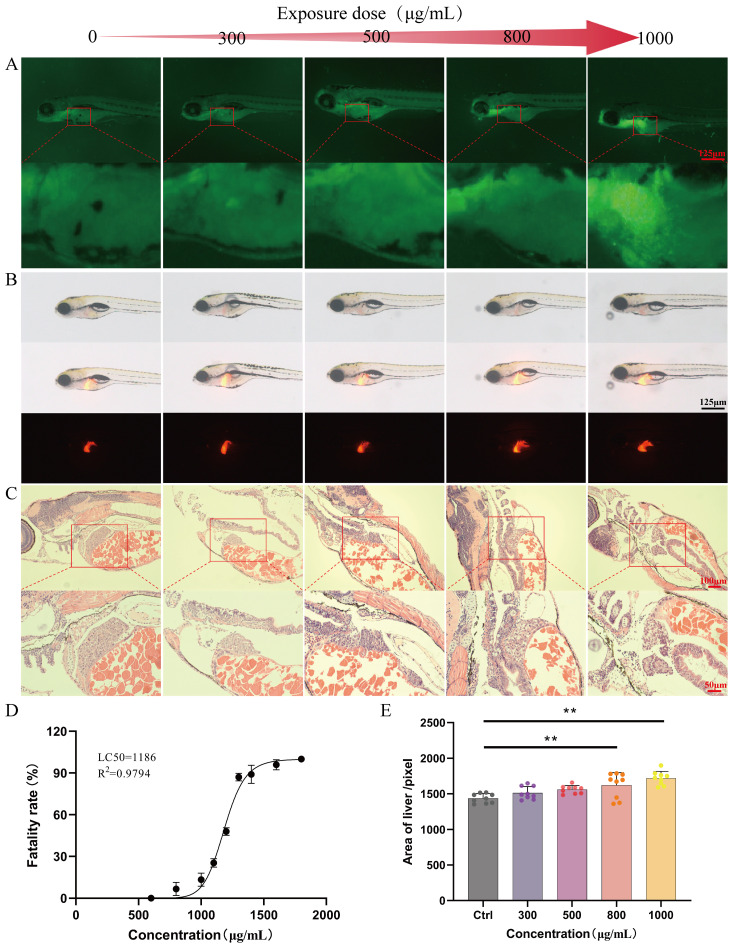
Safety evaluation results **(A)** AO staining (40 ×). **(B)** Evaluation of liver phenotype in zebrafish (40 ×). **(C)** Pathological section of zebrafish liver (20 ×). **(D)** “Dose-mortality” curve of Kushen extract. **(E)** Effects of Kushen extract on liver area of zebrafish. (**p < 0.01).

Based on the statistical analysis of the mortality rate of zebrafish exposed to different concentrations of Kushen extract for 24 hours, the dose-toxicity curve of Kushen extract on zebrafish were constructed (**Figure 2D**). The results indicated that the LC_10_ and LC_0_ values of Kushen extract on zebrafish larvae were 1000 μg·mL^−1^ and 500 μg·mL^−1^ , respectively. The AO staining experiment revealed that, in comparison to the control group, exposure to concentrations of 800 μg·mL^−1^ and 1000 μg·mL^−1^ induced prominent yellow-green fluorescence spots in the liver region of zebrafish, indicating dose-dependent hepatocyte apoptosis. Conversely, no significant hepatocyte apoptosis was observed following exposure to 300 μg·mL^−1^ and 500 μg·mL^−1^ (**Figure 2A**). Furthermore, compared with the control group, only the 800 μg·mL^−1^ and 1000 μg·mL^−1^ exposure groups exhibited a significant increase in liver fluorescence area (*p*< 0.05), while no such increase was observed at concentrations of 300 μg·mL^−1^ and 500 μg·mL^−1^ (*p*> 0.05) (**Figures 2B, E**). Histopathological analysis revealed that the liver tissue structure of zebrafish in the control group was intact, with evenly distributed cytoplasm, the normal nuclear morphology. In contrast, the liver tissue structure of zebrafish in the treated groups were disordered, with the loosely arranged hepatocytes, the reduced cytoplasm. Moreover, these abnormalities were more pronounced in the high-dose group. (**Figure 2C**).

### 3.2 Evaluation of the therapeutic efficacy of Kushen extract in IBD

**Figure 3:**
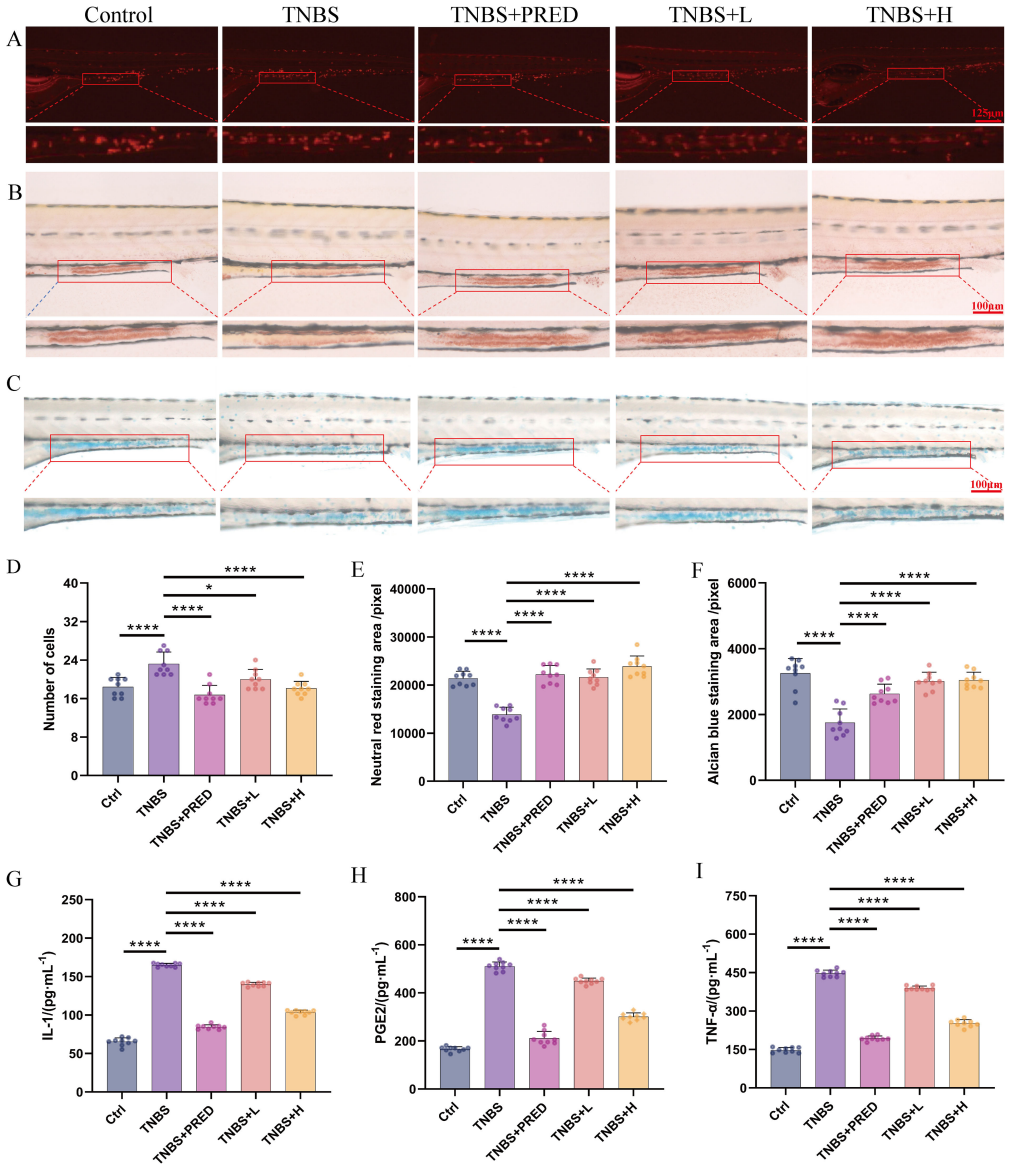
Effectiveness evaluation results **(A)** Neutrophil number changes (80 ×). **(B)** Neutral red staining (160 ×). **(C)** Alcian blue staining(160 ×). **(D)** Changes of neutrophil number. **(E)** Changes in the area stained with neutral red. **(F)** Changes in the area stained with Alcian blue. **(G)** Changes in IL-1β content. **(H)** Changes in PGE2 content. **(I)** Changes in TNF-α content. (*p < 0.05, ****p < 0.0001).

Based on the aforementioned safety evaluation results, exposure concentrations of 500 μg·mL^−1^ (H) and 300 μg·mL^−1^ (L) were selected for subsequent efficacy evaluation experiments. To visually demonstrate the protective effects of Kushen extract on IBD, this study employed a transgenic zebrafish model to assess its impact on intestinal neutrophils, which was considered as an indicator to validate its anti-inflammatory properties. The findings revealed that, compared with the control group, the model group exhibited significant neutrophil aggregation in the zebrafish intestine. In contrast, treatment with 500 μg·mL^−1^ Kushen extract significantly reduced the number of intestinal neutrophils, with statistically significant differences (*p* < 0.05) (**Figures 3A, D**). Furthermore, biochemical analysis indicated that, relative to the control group, levels of IL-1β, TNF-α, and PGE2 were markedly elevated in the model group. However, different concentrations of Kushen extract dose-dependently decreased these inflammatory factors, with all differences being statistically significant (*p* < 0.01) (**Figures 3G–I**). Collectively, these results confirm that Kushen extract exhibits a pronounced inhibitory effect on the inflammatory response in the IBD model.

To further evaluate the impact of Kushen extract on intestinal function, the neutral red staining and alcian blue staining techniques were employed to evaluate the effect of Kushen extract on the endocytic capacity of intestinal cells and the mucin protein secretion ability of goblet cells in zebrafish, respectively. The results demonstrated that, compared with the control group, the areas of neutral red staining and alcian blue staining in the intestinal tract of the model group were significantly decreased (*p* < 0.01). Within the safe dosage range of Kushen extract, both 300 μg·mL^−1^ and 500 μg·mL^−1^ treatment groups effectively reversed the reduction in staining area observed in the model group, with statistically significant differences (*p* < 0.01). Neutral red staining results (**Figures 3B, E**) demonstrate an enhancement in the endocytic activity of intestinal cells in zebrafish following Kushen extract treatment. Likewise, the alcian blue staining area, which was significantly decreased in the model group, was notably improved after administration of both low and high doses of Kushen extract (**Figures 3C, F**). These findings suggest that Kushen extract can markedly enhance the endocytic function of intestinal cells and the mucin protein secretion capability in the model, thereby confirming its substantial protective effect against IBD-related intestinal damage.

### 3.3 Investigation of the mechanism of action of Kushen extract in treating IBD via network pharmacology

**Figure 4:**
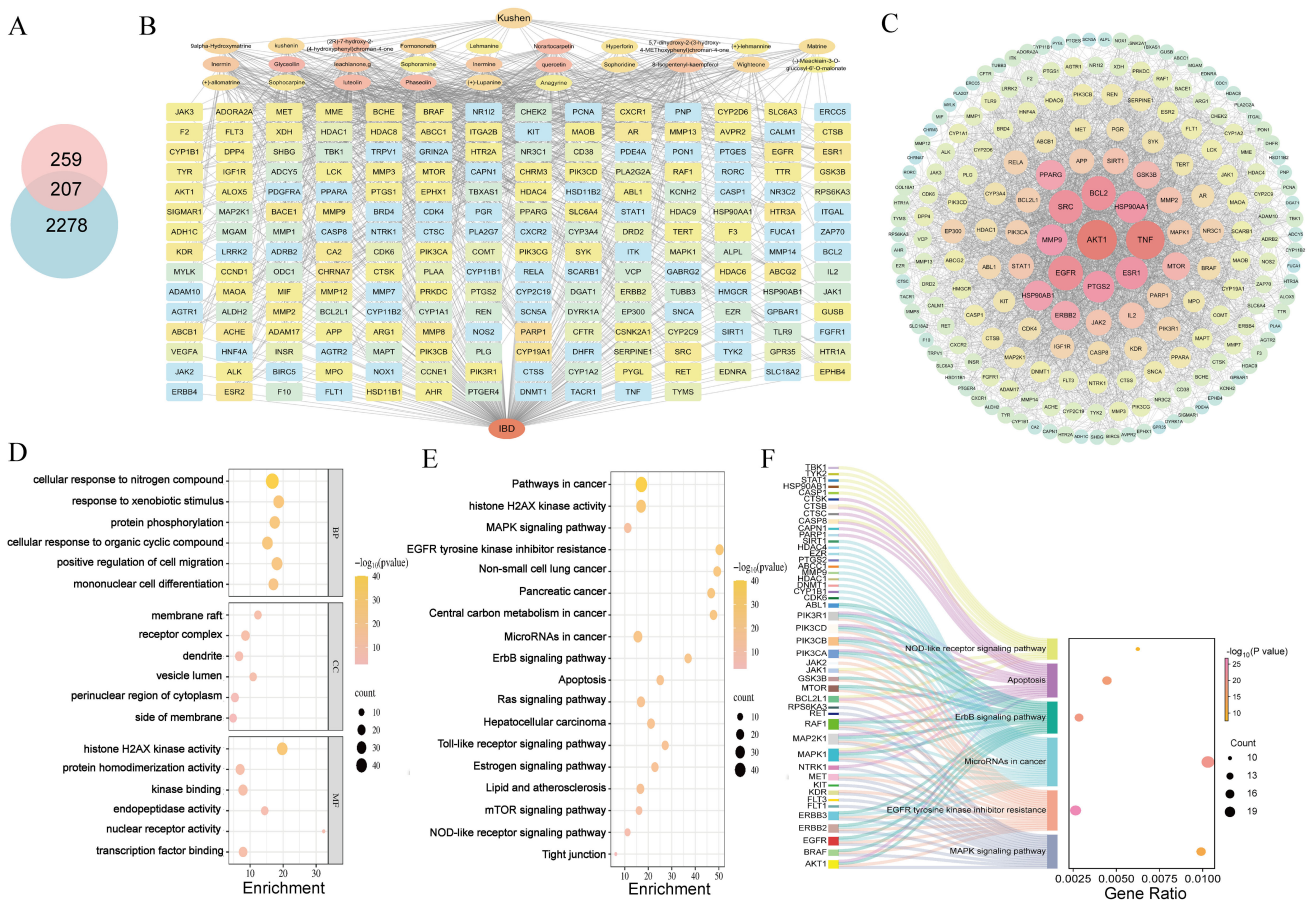
Network pharmacology reveals mechanisms. **(A)** Venn diagram of intersection targets of Kushen extract and IBD. **(B)** “Active ingredients–targets–disease” interaction network. **(C)** PPI network diagram. **(D)** GO enrichment analysis. **(E)** KEGG enrichment analysis. **(F)** Sankey-bubble plot of KEGG enrichment analysis.

Network pharmacology analysis retrieved 466 predicted targets for Kushen extract. Database screening revealed 2,485 disease-related targets associated with IBD. Venn diagram analysis (**Figure 4A**) identified 207 shared targets between Kushen extract and IBD. The “active ingredients-targets-disease” network diagram (**Figure 4B**) demonstrated that bioactive components of Kushen extract may exert therapeutic effects against IBD through multi-target interactions. The shared targets were imported into the STRING database, and protein-protein interaction (PPI) data with confidence scores > 0.4 were selected to construct a network using Cytoscape 3.7.2. Key hub targets with high degree values included Protein kinase B1 (AKT1), Tumor necrosis factor (TNF), Epidermal growth factor receptor (EGFR) and B-cell lymphoma 2 (BCL-2) (**Figure 4C**). GO enrichment analysis revealed 1514 significantly enriched biological process (BP) terms, including “cellular response to nitrogen compound”, “response to xenobiotic stimulus”, and “protein phosphorylation”; 100 cellular component (CC) terms, including “receptor complex”, “membrane raft”, and “vesicle lumen”; and 230 molecular function (MF) terms, including “histone H2AX kinase activity”, “protein serine/threonine kinase activity”, and “kinase binding” (**Figure 4D**). KEGG pathway enrichment analysis identified 175 significantly enriched pathways. The top ten enriched pathways included Apoptosis, MAPK signaling pathway, EGFR tyrosine kinase inhibitor resistance, and Alzheimer disease and so on (**Figures 4E, F**).

### 3.4 Transcriptomics results

**Figure 5:**
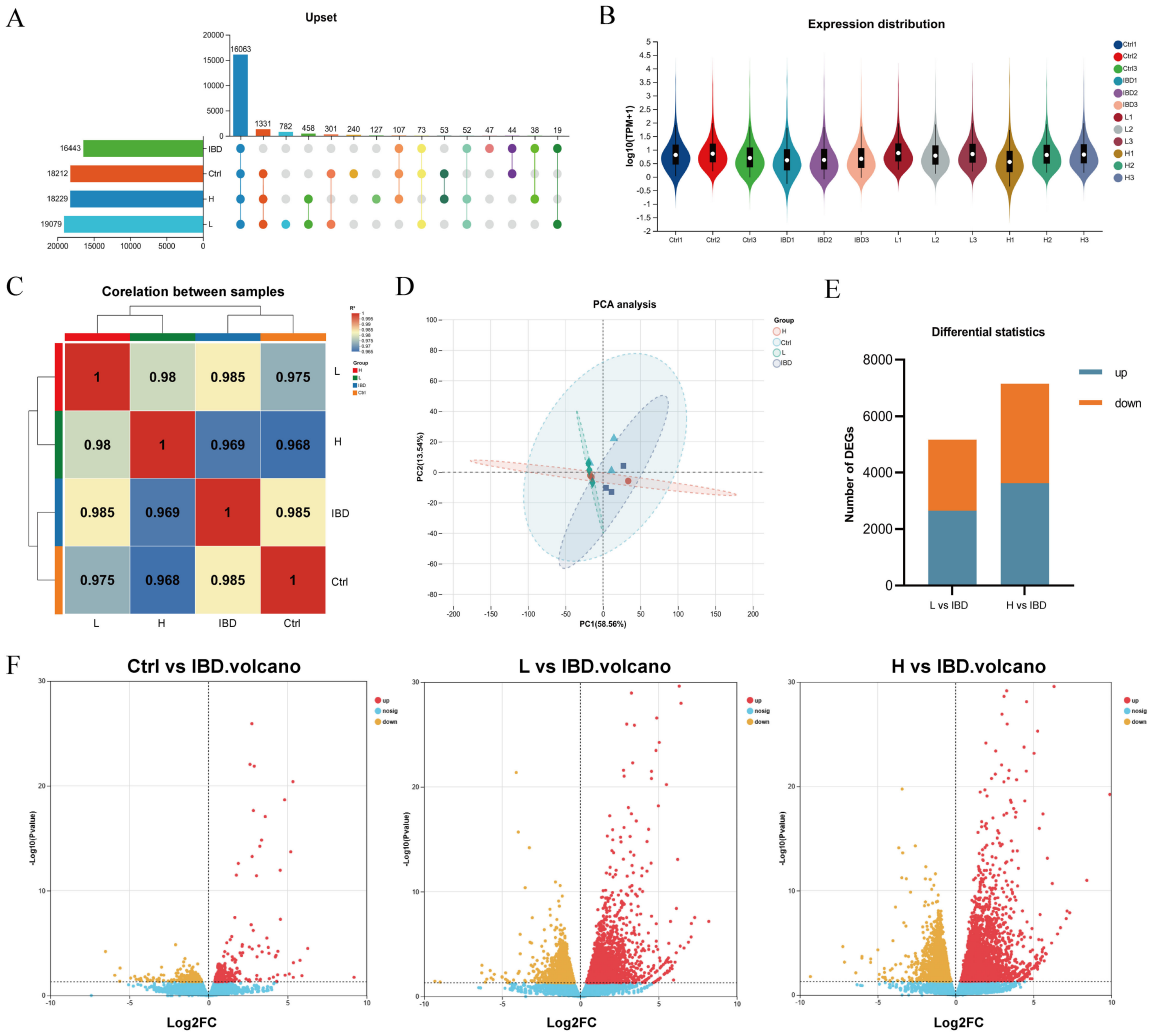
Transcriptomics reveals the mechanism. **(A)** Veen Analysis Upset Chart. **(B)** Sample gene expression distribution. **(C)** Heat map for inter-sample correlation analysis. **(D)** Inter-sample PCA analysis. **(E)** Stacked plot of differential genes. **(F)** Volcano plot showing upregulated and downregulated DEGs after treatment (log2FC≥ 1, *p < 0.05*).

The Venn diagram analysis among samples (**Figure 5A**) clearly illustrated the distribution characteristics of co-expressed genes across different samples and highlighted the quantitative features of specific genes with in each group. In the expression quantity distribution analysis (**Figure 5B**), the variations in expression levels among different samples were elaborated in detail. The inter-sample correlation analysis (**Figure 5C**) comprehensively assessed the consistency of replicate samples, further validating the rationality of the experimental design. The results of principal component analysis (PCA) (**Figure 5D**) demonstrated that samples from different groups exhibited a distinct separation trend at the transcriptome level, which strongly indicated that modeling and drug intervention exerted regulatory effects on the overall transcriptional pattern. Transcriptomic analysis results (**Figures 5E, F**) revealed that compared with the model group, in zebrafish treated with the low dose (300 μg·mL^−1^ ) of Kushen extract exhibited 2631 up-regulated and 2505 down-regulated DEGS; whereas those treated with the high dose (500 μg·mL^−1^ ) demonstrated 3592 DEGs up-regulated and 3499 were down-regulated DEGs, respectively.

**Figure 6:**
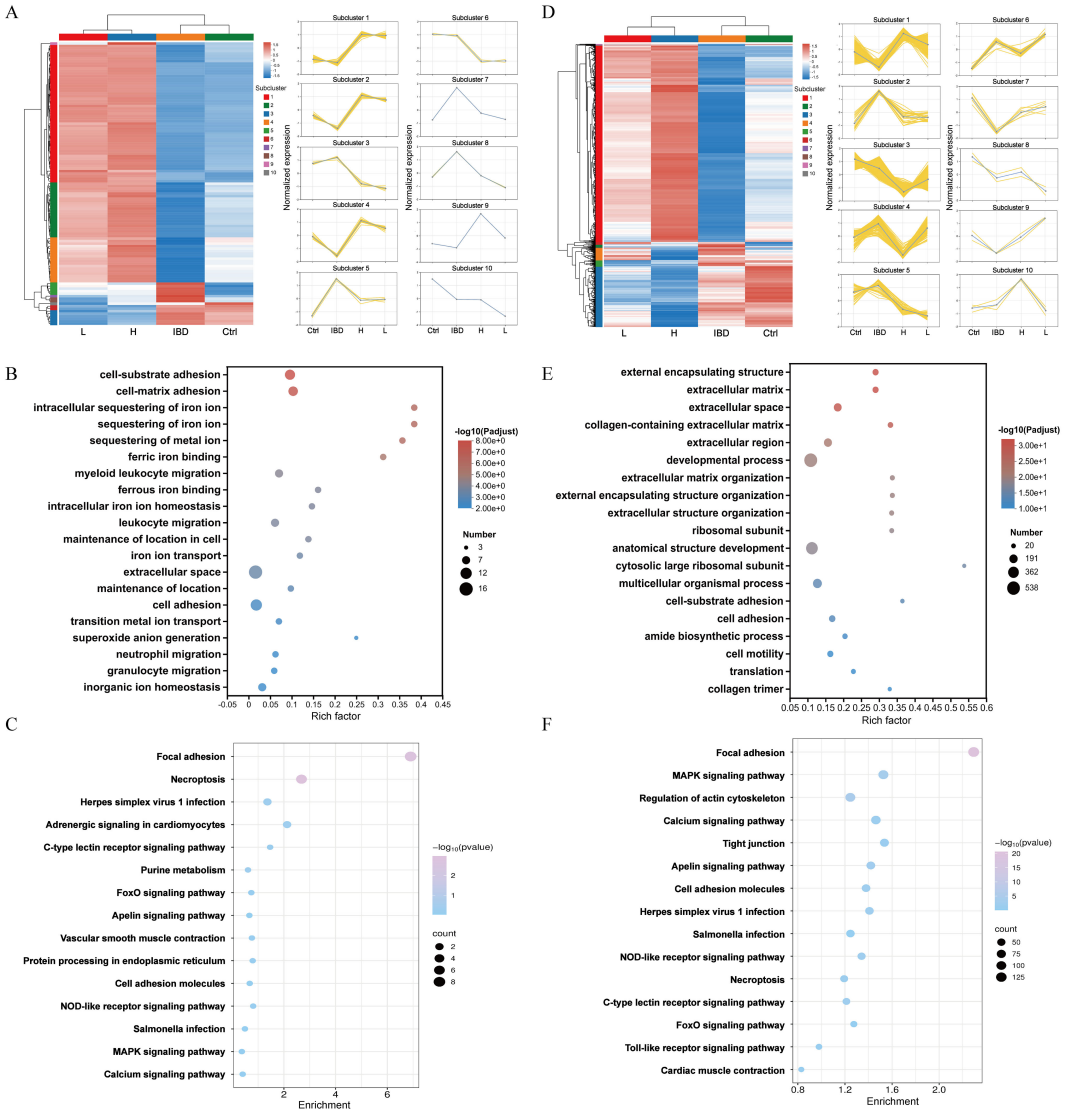
Functional enrichment of genes in zebrafish. **(A)** Heatmap of DEGs in Kushen extract low-dose group (*p*-adjust<0.05, FC≥2). **(B)** GO analysis of DEGs in Kushen extract low-dose group. **(C)** Results of KEGG analysis of DEGs in Kushen extract low-dose group. **(D)** Heatmap of DEGs in Kushen extract high-dose group groups. **(E)** GO analysis of DEGs in Kushen extract high-dose group. **(F)** Results of KEGG analysis of DEGs in Kushen extract high-dose group.

**Figures 6A, D** illustrate the expression level trends of genes across different samples. GO enrichment analysis categorizes results into BP, CC, MF. In the low-dose Kushen extract group, DEGs were most significantly enriched in GO terms related to “external encapsulating structure organization”, “extracellular matrix”, and “extracellular space” (**Figure 6B**). In the high-dose Kushen extract group, DEGs showed significant enrichment in terms associated with “cell-substrate adhesion”, “intracellular sequestering of iron ion”, and “sequestering of metal ion” (**Figure 6E**). KEGG enrichment analysis was also performed on DEGs from the different Kushen extract dose groups to explore the underlying mechanisms. KEGG pathway analysis indicated that pathways such as “Calcium signaling pathway”, “MAPK signaling pathway”, and “Cell adhesion molecules” were notably affected in the low-dose group, while pathways including “Calcium signaling pathway”, “Tight junction”, and “Apoptosis” were prominent in the high-dose group (**Figures 6C, F**). These results suggest that Kushen extract may exert its therapeutic effects on IBD by modulating pathways related to calcium signaling and apoptosis, among others.

### 3.5 Integrated network pharmacology and transcriptomics analysis

**Figure 7:**
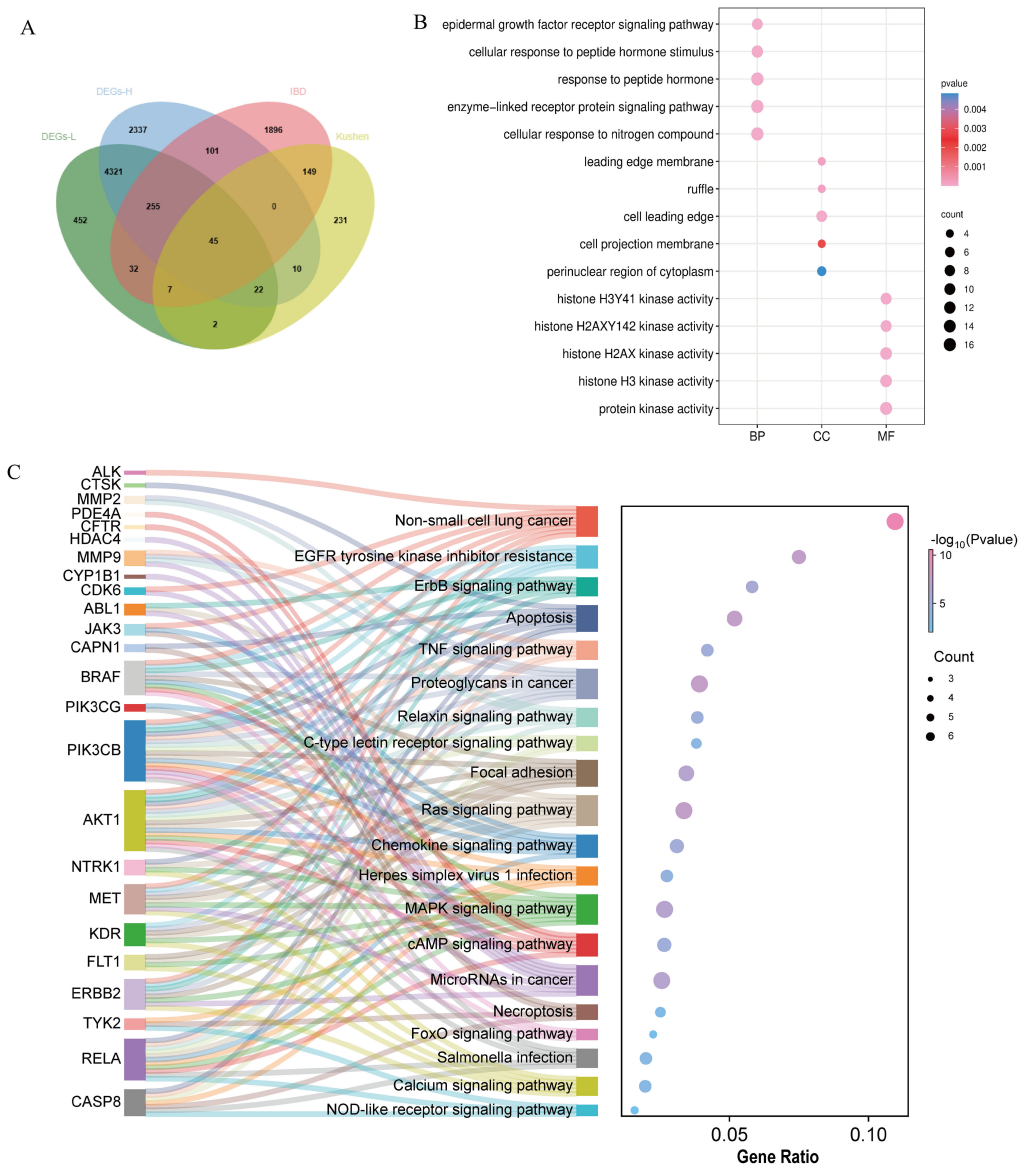
Integrated network pharmacology and transcriptomics analysis. **(A)** Venn diagram of the intersection between network pharmacology and transcriptomics. **(B)** GO analysis of the combination of network pharmacology and transcriptomics. **(C)** KEGG analysis of the combination of network pharmacology and transcriptomics.

To better elucidate the potential mechanism of Kushen extract in treating IBD, we performed an intersection analysis between the DEGs from the transcriptomics analysis (low-dose Kushen extract group vs. IBD group and high-dose Kushen extract group vs. IBD group) and the targets identified by network pharmacology analysis, resulting in overlapping DEGs (**Figure 7A**). Subsequently, GO and KEGG enrichment analyses were conducted on these overlapping targets (**Figures 7B, C**). The results revealed that the “Calcium signaling pathway”, “Apoptosis”, “FoxO (Forkhead Box O) signaling pathway”, and “MAPK signaling pathway” were among the most significantly enriched pathways.

### 3.6 Key DEGs in RT-qPCR validation and molecular docking

**Figure 8:**
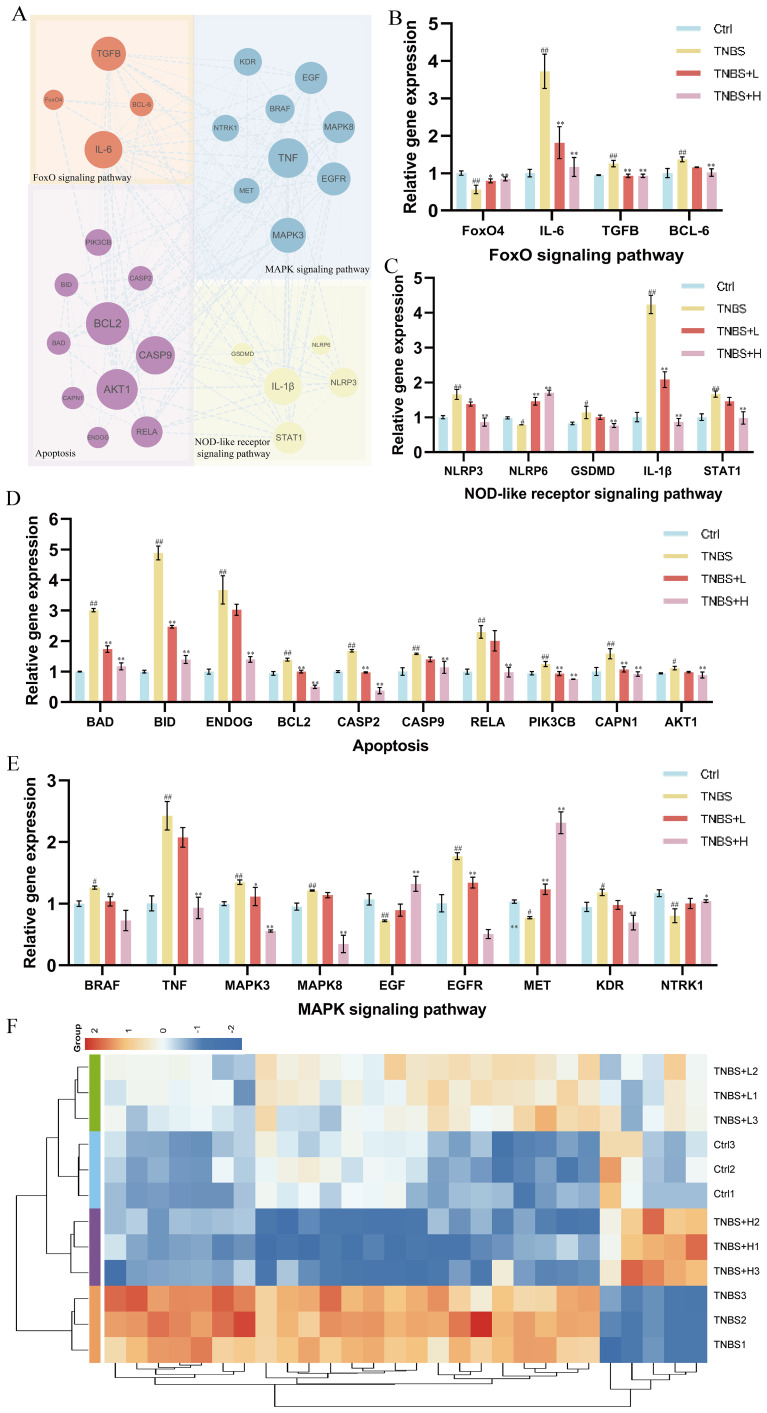
RT-qPCR validation of gene expression. **(A)** PPI network diagram of FoxO4/NOD-like/Apoptosis and MAPK signaling pathways relative genes. **(B)** Gene expression of the FoxO signaling pathway. **(C)** Gene expression of the NOD-like receptor signaling pathway. **(D)** Gene expression of the Apoptosis. **(E)** Gene expression of the MAPK signaling pathway. **(F)** Heat map of gene expression levels. (*p < 0.05, **p < 0.01).

**Figure 9:**
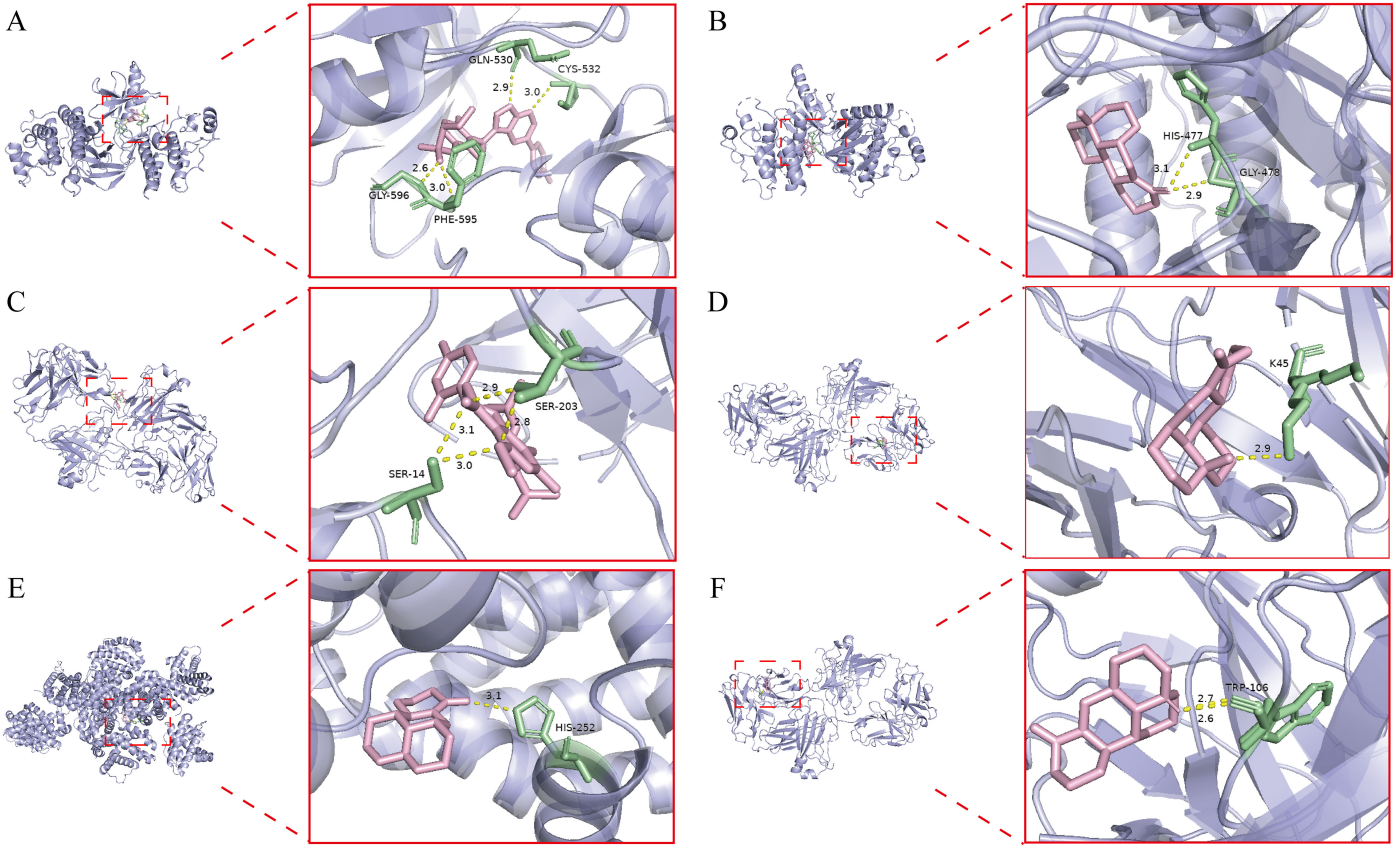
Molecular docking result. **(A)** Kurarinone-BRAF. **(B)** Oxysophoridine-BRAF. **(C)** Kurarinone-EGFR. **(D)** Oxymatrine-EGFR. **(E)** Matrine- BCL2. **(F)** Sophoridine-EGFR.

Based on the integrated network pharmacology and transcriptomics analysis, we identified key pathways and targets underlying Kushen extract’s therapeutic effect on IBD (**Figure 8A**). The expression levels of genes associated with these identified pathways were further assessed by RT-qPCR. The results demonstrated significant modulation in the FoxO signaling pathway, apoptosis, NOD-like receptor signaling pathway, and MAPK signaling pathway. The expression levels of several key genes, including FoxO4, NLRP6, EGF, MET, and NTRK1 were decreased in the model group compared to the control group (*p* < 0.05). Administration of Kushen extract was found to upregulate the expression of these genes. Conversely, an opposite expression trend, which was significantly different observed for the remaining genes examined (**Figures 8B–E**). For enhanced clarity and visualization, these results are also presented as a heatmap (**Figure 8F**). To provide a structural basis for these observed gene expression changes, molecular docking analyses were performed. The molecular docking analysis demonstrated stable binding conformations between the key constituents of Kushen extract and their corresponding target proteins, as illustrated in **Figure 9**. As shown in **Supplementary Table 2**, the evaluated protein-ligand complexes displayed favorable binding energies, suggesting strong binding affinity. Notably, oxymatrine exhibited the most potent interaction with EGFR, evidenced by a significantly low binding energy value. This high-affinity binding is stabilized by specific hydrogen bonding interactions and hydrophobic forces.

## 4 Discussion

Collectively, this study elucidates a critical dose-dependent duality of Kushen extract in managing IBD using a zebrafish model. Within the defined therapeutic window (≤500 μg·mL^−1^ ), the extract exhibited significant anti-inflammatory effects, including reduced neutrophil infiltration, restoration of intestinal phagocytic function, and normalization of pro-inflammatory cytokine levels, with no detectable hepatotoxicity. In contrast, supra-therapeutic doses (>800 μg·mL^−1^ ) resulted in pronounced hepatic damage characterized by increased apoptosis and structural disruption. This clear dosage distinction highlights the essential need for precise clinical dosing to balance therapeutic efficacy with potential toxicity.

**Figure 10:**
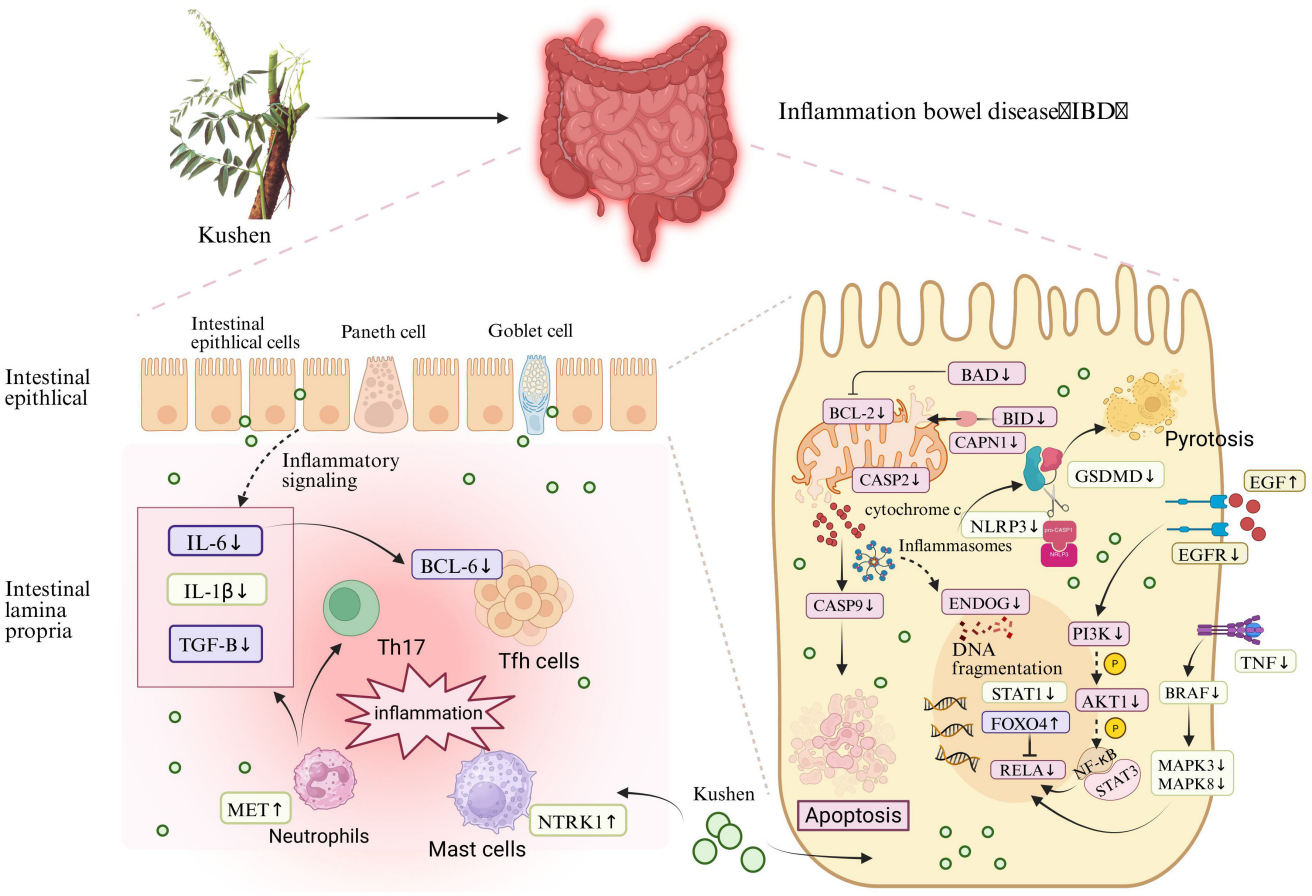
Mechanism of Kushen extract in treating IBD.

From a mechanistic perspective, the observed bioactivity is associated with the coordinated regulation of key signaling pathways. These pathways were consistently identified through integrated network and transcriptomic analyses and were further confirmed through functional validation. We assessed the expression of several critical genes within four essential signaling pathways—FoxO signaling, NOD-like receptor signaling, apoptosis, and MAPK signaling—following treatment with Kushen extract in an IBD model (**Figure 10**). The resulting expression profiles indicated that Kushen extract exerts diverse protective effects via immune modulation, suppression of inflammation, epithelial barrier protection, and promotion of tissue repair.

The FoxO signaling pathway serves as a critical regulator in maintaining intestinal immune homeostasis and fine-tuning inflammatory responses in IBD. Among its key components, FoxO4 functions as a crucial transcription factor that modulates immune activity and has demonstrated protective effects in experimental models of colitis. It exerts its influence by directly inhibiting the transcriptional activity of NF-κB p65, thereby suppressing the expression of pro-inflammatory cytokines such as tumor necrosis factor-alpha (TNF-α) and interleukin-6 (IL-6), which contributes to an overall anti-inflammatory effect (34). Clinical evidence further supports this mechanism, showing that patients with ulcerative colitis (UC) in remission exhibit significantly higher levels of FoxO4 expression compared to those with active disease, with a positive correlation observed between FoxO4 expression and histological healing. The FoxO signaling pathway regulates pivotal mediators involved in IBD pathogenesis, including IL-6, which plays a dual role as both a biomarker and a driver of chronic intestinal inflammation. This gene encodes a cytokine that plays a role in B-cell-mediated inflammation and maturation. It is predominantly produced at acute and chronic inflammatory sites, where it is secreted into the bloodstream and elicits inflammatory responses via interaction with the interleukin-6 receptor α. Transforming growth factor beta-1 (TGF-β) is an anti-inflammatory cytokine involved in immune regulation, epithelial tissue repair, and fibrotic processes. It is expressed by colonic epithelial cells and lamina propria cells, with increased levels observed in tissues from individuals with IBD, particularly in ulcerated and regenerating areas (35). In the context of IBD, TGF-β promotes Th17 cell differentiation when co-expressed with IL-6 under pro-inflammatory conditions (36). Moreover, intestinal epithelial cells are capable of secreting extracellular vesicles containing TGF-β (37). The elevated expression of TGF-β in IBD tissues may represent a compensatory mechanism aimed at counteracting ongoing inflammation. T follicular helper (Tfh) cells constitute a distinct subset of CD4+ T cells. Additionally, B-cell lymphoma 6 (BCL-6) serves as a critical transcription factor required for the differentiation of Tfh cells (38). Research has demonstrated that the IL-6/IL-6R signaling axis primarily mediates BCL-6 expression through the activation of STAT1 and STAT3, thereby amplifying inflammatory responses (39). Kushen extract has been shown to modulate the aforementioned aberrant gene expressions in a regulatory, opposing manner. Collectively, these synergistic effects suggest that Kushen extract contributes to the restoration of mucosal immune homeostasis and the suppression of chronic intestinal inflammation.

The NOD-like receptor signaling pathway serves as a critical element of the innate immune system, functioning in the recognition of microbial threats and cellular stress. In IBD, dysfunction within this pathway contributes to persistent inflammation and disruption of the epithelial barrier. The NOD-like receptor family pyrin domain-containing 3 (NLRP3) acts as a central sensor that, upon detection of damage-associated molecular patterns (DAMPs), assembles an inflammasome complex together with apoptosis-associated speck-like protein containing a CARD (ASC) and pro-caspase-1 (proCASP1) (40). This process facilitates the activation of caspase-1 (CASP1), which subsequently cleaves interleukin-1β (IL-1β) and gasdermin D (GSDMD), thereby initiating cytokine secretion and pyroptotic cell death (41, 42). IL-1β is a potent pro-inflammatory cytokine that promotes neutrophil infiltration, fibroblast activation, and epithelial damage within the mucosa of IBD patients (43). GSDMD serves as a key executor of inflammasome-dependent pyroptosis and various downstream immune responses. Upon activation of the inflammasome, the N-terminal fragment of GSDMD forms pores in the cell membrane, facilitating the secretion of cytokines and initiating pyroptotic cell death (44). In IBD, GSDMD is highly expressed in intestinal epithelial cells and has been shown to mediate the release of extracellular vesicles containing IL-1β from these cells (45). In contrast to NLRP3, which promotes inflammation, NLRP6 exerts protective effects by preserving epithelial barrier integrity, limiting microbial translocation, and enhancing mucin production (46, 47). Furthermore, NLRP6 contributes to the regulation of gut microbiota composition and supports IL-18–mediated tissue repair processes (48, 49). Signal transducer and activator of transcription 1 (STAT1), which is activated downstream of cytokine signaling pathways, enhances the transcription of pro-inflammatory genes and promotes the differentiation of Th1 and Th17 cells, thereby exacerbating IBD progression (50). The therapeutic effects of Kushen have been found to counteract pathological changes induced by these inflammatory pathways. Specifically, Kushen effectively suppresses NLRP3-driven inflammatory responses, potentially restores NLRP6-mediated protective mechanisms, and modulates STAT1-dependent immune cell differentiation, ultimately alleviating intestinal inflammation and promoting mucosal healing.

In IBD, excessive activation of the apoptosis signaling pathway contributes to impaired epithelial barrier function, increased immune cell infiltration, and persistent mucosal inflammation. Among the upstream regulators of mitochondrial apoptosis, BAD (BCL-2-associated death promoter) and BID (BH3-interacting domain death inducer) are two key pro-apoptotic members of the Bcl-2 family (51). These proteins promote apoptosis by compromising mitochondrial membrane integrity and facilitating the release of cytochrome c (52). Specifically, BAD exerts its apoptotic effect by binding to and neutralizing anti-apoptotic proteins such as BCL2. BID, upon cleavage by calpain—which is encoded by CAPN1—translocates to the mitochondria, where it interacts with Bax/Bak (BCL2 antagonist/killer) to enhance membrane permeability and promote cytochrome c release (53). BCL2 interacts with pro-apoptotic proteins, thereby preventing mitochondrial outer membrane permeabilization, suppressing the release of cytochrome c, and blocking subsequent caspase activation. In IBD, these molecules are commonly upregulated, resulting in increased epithelial cell death and mucosal injury (54). Specifically, the expression levels of BAD, BID, CAPN1, and BCL2 are frequently elevated in IBD, contributing to excessive apoptosis of epithelial cells and consequent mucosal damage. The downregulation of BAD, BID, and CAPN1 following treatment with Kushen suggests a suppression of early mitochondrial apoptotic signals, which may indicate enhanced preservation of the epithelial layer. CASP2 and CASP9 function as initiator caspases within the intrinsic apoptotic pathway (55, 56). CASP9 is activated by the apoptosome complex following the release of cytochrome c from mitochondria, whereas CASP2 is triggered by cellular stress independently of mitochondrial involvement (57, 58). Both caspases initiate the proteolytic cascade that leads to the activation of effector caspases and ultimately results in programmed cell death. The observed reduction in the expression of CASP2 and CASP9 after Kushen administration implies an inhibition of apoptotic signaling, potentially offering protection against excessive epithelial turnover in the inflamed intestine. ENDOG (endonuclease G) is another pro-apoptotic molecule that is released from mitochondria during apoptosis (59). Following mitochondrial outer membrane permeabilization, ENDOG translocates into the nucleus, where it promotes chromatin condensation and DNA fragmentation (60). RELA, the p65 subunit of NF-κB, functions at the intersection of inflammatory and apoptotic signaling pathways. In IBD, RELA is commonly overactivated, thereby promoting the transcription of both pro-inflammatory mediators and genes associated with cell survival (61, 62). The suppression of RELA by Kushen implies a reduction in chronic inflammation and apoptotic signaling, which aligns with the observed decrease in mucosal damage. Phosphoinositide 3-kinase (PI3K) recruits and activates its downstream effector AKT1 upon stimulation by extracellular signals. Activated AKT1 initiates multiple signaling cascades through phosphorylation, including the activation of the NF-κB pathway, which facilitates the nuclear translocation of NF-κB and enables it to induce the transcription of pro-inflammatory cytokines such as TNF-α and IL-1β (63). This mechanism contributes to the hallmark inflammatory microenvironment and cytokine dysregulation characteristic of IBD. The downregulation of PIK3CB and AKT1 expression following Kushen treatment indicates an inhibition of this critical pro-inflammatory signaling axis, thereby aiding in the mitigation of inflammatory responses.

The MAPK signaling pathway is critical for transducing extracellular inflammatory signals into intracellular responses and plays a central role in regulating immune responses, epithelial repair, and inflammatory processes in IBD (64). BRAF (B-Raf proto-oncogene, serine/threonine kinase) functions as a key upstream activator of the MEK-ERK cascade, promoting the phosphorylation of downstream effectors such as MAPK3 and MAPK8. These kinases regulate the transcription of inflammatory genes, and their hyperactivation in IBD has been associated with increased epithelial cell apoptosis and persistent mucosal inflammation (65). TNF is a central pro-inflammatory cytokine that enhances activation of the MAPK pathway via TNF receptor signaling, further stimulating MAPK3 and MAPK8 activity (66). This results in excessive production of inflammatory mediators, disruption of the intestinal epithelial barrier, and exacerbation of disease severity. The observed downregulation of TNF, BRAF, MAPK3, and MAPK8 following Kushen treatment indicates effective suppression of this inflammatory signaling axis. EGF (Epidermal Growth Factor) is a crucial growth factor involved in promoting intestinal epithelial cell proliferation and facilitating barrier repair (67). In the context of IBD, reduced expression of EGF contributes to impaired mucosal healing and compromised barrier integrity. Restoration of EGF levels has been shown to enhance tight junction stability and promote epithelial regeneration, thereby offering protection against mucosal damage (68, 69). EGFR (Epidermal Growth Factor Receptor), the receptor for EGF, is a transmembrane tyrosine kinase receptor that, upon activation, initiates downstream signaling cascades such as the MAPK/ERK and PI3K/AKT pathways, which are essential for cell survival, proliferation, and anti-apoptotic effects (70, 71). MET (Mesenchymal-Epithelial Transition factor) is a receptor tyrosine kinase activated by HGF (hepatocyte growth factor). Studies (72) have demonstrated that HGF-MET signaling is activated in neutrophils, promoting chemotaxis and cytotoxicity, enhancing IL-1β production, inducing Th17 cell expansion, and exacerbating intestinal inflammation. KDR, also known as VEGFR-2, is a primary receptor for VEGF-A, mediating abnormal angiogenesis, increased vascular permeability, and enhanced immune cell infiltration, all of which contribute to mucosal inflammation (73). Downregulation of KDR following treatment with Kushen suggests that inhibition of the VEGF-A/KDR signaling pathway may help mitigate vascular and immune dysfunction, thereby alleviating inflammation and facilitating mucosal healing in IBD. NTRK1 (neurotrophic receptor tyrosine kinase 1) encodes TrkA, a high-affinity receptor for nerve growth factor (NGF), which is upregulated in IBD, particularly in mast cells and lamina propria monocytes. Overexpression of TrkA enhances the release of inflammatory mediators and activates immune cells, thus contributing to neurogenic inflammation and mucosal damage (74, 75).

While the present study offers novel insights into the dose-dependent efficacy and safety of Kushen extract in a zebrafish model of IBD, several limitations must be acknowledged. First, the inherent variability in the composition of natural products across different batches may affect the reproducibility and stability of the observed dose-effect relationship. Second, our mechanistic analysis primarily relies on transcriptional data obtained through RT-qPCR, with a lack of corresponding protein-level evidence to substantiate post-transcriptional regulation. This limitation impedes the definitive establishment of causal relationships between Kushen extract treatment and the modulation of key signaling pathways. Furthermore, the study did not include functional validation via pathway-specific inhibition (e.g., through the use of MAPK inhibitors), which would have provided stronger evidence linking the identified pathways to the therapeutic effects of Kushen extract. Lastly, although zebrafish exhibit conserved inflammatory pathways and share key intestinal structural features with mammals, this model does not fully recapitulate the complexity of human IBD, which arises from intricate interactions among genetic predisposition, gut microbiota, and environmental influences. These limitations may hinder the direct translation of our findings to clinical applications. We emphasize that our results should be interpreted as preliminary evidence, and further validation through mammalian models and clinical studies is essential to confirm therapeutic efficacy and establish optimal dosage regimens.

To address these limitations, future studies will focus on several key directions: validating the therapeutic efficacy and safety of Kushen extract in murine models of IBD, which more closely mimic human pathological features, such as gut microbiota dysregulation and adaptive immune responses; supplementing the current findings with protein-level analyses and functional experiments to further elucidate the underlying mechanisms; investigating the individual contributions of major Kushen extract constituents to hepatotoxicity and anti-inflammatory effects in order to clarify the material basis of its dual pharmacological properties; and establishing standardized quantification methods for key alkaloids and flavonoids across multiple batches to confirm the consistency and generalizability of our findings. These efforts will not only strengthen the validity of our conclusions but also provide a more solid foundation for the clinical application of Kushen extract-based therapies in the management of IBD.

## 5 Conclusion

This study comprehensively assessed the safety and efficacy of Kushen extract using a zebrafish model. Supra-therapeutic doses (> 800 μg·mL^−1^ ) induced significant hepatotoxicity as evidenced by hepatic morphological abnormalities and increased hepatocyte apoptosis. In contrast, administration within the safe dosage range (≤ 500 μg·mL^−1^ ) did not exhibit notable toxicity but conferred therapeutic benefits against TNBS-induced IBD. Specifically, Kushen extract treatment reduced intestinal neutrophil infiltration, lowered levels of key pro-inflammatory cytokines (IL-1β, TNF-α, PGE2), and improved intestinal phagocytic function and goblet cell mucin secretion.

Beyond these findings, this work demonstrates important innovation. Unlike previous studies that emphasized only pharmacological activity, we systematically examined the dose-dependent duality of efficacy and hepatotoxicity, thereby defining a clearer safety–efficacy window. By integrating network pharmacology, transcriptomic analysis, RT-qPCR and molecular docking, we combined predictive analysis with experimental confirmation to demonstrate that the therapeutic effects of Kushen extract are primarily achieved through the interaction between multiple key active components and FoxO4, NOD-like receptor, apoptosis, and MAPK signaling pathways. This integrative approach also enabled the identification of potential risks associated with supra-therapeutic exposure. Together, these results provide experimental evidence that deepens understanding of Kushen extract’s molecular actions and offer a basis for its further clinical translation and the development of safer Kushen extract-derived agents. The established therapeutic window in zebrafish models offers a foundational reference for subsequent preclinical development, and the correlation between effective dosage and traditional application practices underscores its potential translational significance. Further investigation in mammalian systems and clinical trials will be necessary to refine dosing recommendations and validate therapeutic efficacy.

## Data Availability

The original contributions presented in the study are included in the article/**Supplementary Material**. Further inquiries can be directed to the corresponding authors.

## References

[1] Sun Z Ye J Sun W Jiang L Shan B Zhang M . Cooperation of TRADD- and RIPK1-dependent cell death pathways in maintaining intestinal homeostasis. Nat Commun. (2025) 16:1890. doi: 10.1038/s41467-025-57211-z, PMID: 39987261 PMC11846980

[2] Blesl A Binder L Halwachs B Baumann-Durchschein F Fürst S Constantini-Kump P . The fecal microbiome of IBD patients is less divertible by bowel preparation compared to healthy controls: results from a prospective study. Inflammation Bowel Dis. (2025) 31:2007–18. doi: 10.1093/ibd/izaf053, PMID: 40296371 PMC12235133

[3] Huang H Fang M Jostins L Umićević Mirkov M Boucher G Anderson CA . Fine-mapping inflammatory bowel disease loci to single-variant resolution. Nature. (2017) 547:173–8. doi: 10.1038/nature22969, PMID: 28658209 PMC5511510

[4] Yin Q da Silva AC Zorrilla F Almeida AS Patil KR Almeida A . Ecological dynamics of Enterobacteriaceae in the human gut microbiome across global populations. Nat Microbiol. (2025) 10:541–53. doi: 10.1038/s41564-024-01912-6, PMID: 39794474 PMC11790488

[5] Torres J Mehandru S Colombel JF Peyrin-Biroulet L . Crohn's disease. Lancet. (2017) 389:1741–55. doi: 10.1016/S0140-6736(16)31711-1, PMID: 27914655

[6] Gorospe J Windsor J Hracs L Coward S Buie M Quan J . Trends in inflammatory bowel disease incidence and prevalence across epidemiologic stages: a global systematic review with meta-analysis. Inflammation Bowel Dis. (2024) 30:S00. doi: 10.1093/ibd/izae020.085

[7] Le Berre C Honap S Peyrin-Biroulet L . Ulcerative colitis. Lancet. (2023) 402:571–84. doi: 10.1016/S0140-6736(23)00966-2, PMID: 37573077

[8] He X Fang J Huang L Wang J Huang X . Sophora flavescens Ait.: Traditional usage, phytochemistry and pharmacology of an important traditional Chinese medicine. J Ethnopharmacol. (2015) 172:10–29. doi: 10.1016/j.jep.2015.06.010, PMID: 26087234

[9] Kim CS Park SN Ahn SJ Seo YW Lee YJ Lim YK . Antimicrobial effect of sophoraflavanone G isolated from Sophora flavescens against mutans streptococci. Anaerobe. (2013) 19:17–21. doi: 10.1016/j.anaerobe.2012.11.003, PMID: 23178373

[10] Yang JM Ip SP Xian Y Zhao M Lin ZX Yeung JHK . Impact of the herbal medicine sophora flavescens on the oral pharmacokinetics of indinavir in rats: the involvement of CYP3A and P-glycoprotein. PloS One. (2012) 7:e31312. doi: 10.1371/journal.pone.0031312, PMID: 22359586 PMC3281083

[11] National Pharmacopoeia Commission . Pharmacopoeia of the people's republic of China. One. Beijing: China Medical Science and Technology Press (2020).

[12] Kim JS Shin SJ Kim JN Kwon MJ Lim EY Kim YT . Radix Sophorae Flavescentis inhibits proliferation and induces apoptosis of AGS human gastric cancer cells. Mol Med Rep. (2019) 19:1911–8. doi: 10.3892/mmr.2018.9776, PMID: 30569168

[13] Fang R Wu R Zuo Q Yin R Zhang C Wang C . Sophora flavescens containing-QYJD formula activates Nrf2 anti-oxidant response, blocks cellular transformation and protects against DSS-induced colitis in mouse model. Am J Chin Med. (2018) 46:1609–23. doi: 10.1142/S0192415X18500829, PMID: 30284461 PMC8111688

[14] Zhu T Zhou D Zhang Z Long L Liu Y Fan Q . Analgesic and antipruritic effects of oxymatrine sustained-release microgel cream in a mouse model of inflammatory itch and pain. Eur J Pharm Sci. (2020) 141:105110. doi: 10.1016/j.ejps.2019.105110, PMID: 31654757

[15] Chen L Shao J Luo Y Zhao L Zhao K Gao Y . An integrated metabolism *in vivo* analysis and network pharmacology in UC rats reveal anti-ulcerative colitis effects from Sophora flavescens EtOAc extract. J Pharm BioMed Anal. (2020) 186:113306. doi: 10.1016/j.jpba.2020.113306, PMID: 32371325

[16] Chen M Ding Y Tong Z . Efficacy and safety of Sophora flavescens (Kushen) based traditional Chinese medicine in the treatment of ulcerative colitis, clinical evidence and potential mechanisms. Front Pharmacol. (2020) 11:603476. doi: 10.3389/fphar.2020.603476, PMID: 33362558 PMC7758483

[17] Hou WB Sun WJ Zhang XW Li YX Zheng YY Sun YX . Five-flavor sophora flavescens enteric-coated capsules for ulcerative colitis: A systematic review and meta-analysis of randomized clinical trials. Evid Based Complement Alternat Med. (2022) 2022:9633048. doi: 10.1155/2022/9633048, PMID: 35069773 PMC8769833

[18] Gao F Deng S Liu Y Wu P Huang L Zhu F . Compound sophora decoction alleviates ulcerative colitis by regulating macrophage polarization through cGAS inhibition: network pharmacology and experimental validation. Aging. (2024) 16:6921–36. doi: 10.18632/aging.205734, PMID: 38613801 PMC11087132

[19] Li L Li J . Shennong bencao jing. Wuhan, China: Hubei Science and Technology Press (2016).

[20] Liu J Zhao Y Xia J Qiu M . Matrine induces toxicity in mouse liver cells through an ROS-dependent mechanism. Res Vet Sci. (2020) 132:308–11. doi: 10.1016/j.rvsc.2020.07.006, PMID: 32717422

[21] Tao X Zhang W Chen L Lu S Li Z Gao Y . The DHCR7 is the key target of lipotoxic liver injury caused by matrine through abnormal activation of the cholesterol synthesis pathway. Toxicon. (2025) 260:108366. doi: 10.1016/j.toxicon.2025.108366, PMID: 40250732

[22] Cui M Zhang Y Tang Y Fan Q Chen X Li J . Hepatotoxicity of Phytolacca acinosa Roxb mediated by phytolaccagenin via ferroptosis/PPAR/P53/ arachidonic acid metabolism. Phytomedicine. (2025) 138:156433. doi: 10.1016/j.phymed.2025.156433, PMID: 39892312

[23] Fan Q Liang R Chen M Li Z Tao X Ren H . Metabolic characteristics of evodiamine were associated with its hepatotoxicity via PPAR/PI3K/AKT/NF-кB/tight junction pathway-mediated apoptosis in zebrafish. Ecotoxicol Environ Saf. (2024) 279:116448. doi: 10.1016/j.ecoenv.2024.116448, PMID: 38754199

[24] Guo S Zhang X Zhang Y Chen X Zhang Y Cao B . Development of a rapid zebrafish model for lead poisoning research and drugs screening. Chemosphere. (2023) 345:140561. doi: 10.1016/j.chemosphere.2023.140561, PMID: 39491111

[25] MacRae CA Peterson RT . Zebrafish as tools for drug discovery. Nat Rev Drug Discov. (2015) 14:721–31. doi: 10.1038/nrd4627, PMID: 26361349

[26] MacRae CA Peterson RT . Zebrafish as a mainstream model for *in vivo* systems pharmacology and toxicology. Annu Rev Pharmacol Toxicol. (2023) 63:43–64. doi: 10.1146/annurev-pharmtox-051421-105617, PMID: 36151053

[27] Lavorato M Iadarola D Remes C Kaur P Broxton C Mathew ND . dldhcri3 zebrafish exhibit altered mitochondrial ultrastructure, morphology, and dysfunction partially rescued by probucol or thiamine. JCI Insight. (2024) 9:e178973. doi: 10.1172/jci.insight.178973, PMID: 39163131 PMC11457866

[28] Sheng Y Li X Ye X Fan Q Li J Qiao C . Integrated transcriptomic and proteomic analysis of hepatotoxic effects of Venenum Bufonis in zebrafish. J Ethnopharmacol. (2025) 348:119865. doi: 10.1016/j.jep.2025.119865, PMID: 40274029

[29] Wang W Li J Zhou Q . The biological function of cytoplasm-translocated ENDOG (endonuclease G). Autophagy. (2024) 20:445–7. doi: 10.1080/15548627.2023.2271750, PMID: 37889084 PMC10813634

[30] Lu S Huang J Zhang J Wu C Huang Z Tao X . The anti-hepatocellular carcinoma effect of Aidi injection was related to the synergistic action of cantharidin, formononetin, and isofraxidin through BIRC5, FEN1, and EGFR. J Ethnopharmacol. (2024) 319:117209. doi: 10.1016/j.jep.2023.117209, PMID: 37757991

[31] Chen M Zhao C Fan Q Li Z Lu S Tao X . Investigation of the applicability of the zebrafish model for the evaluation of aristolochic acid-related nephrotoxicity. Phytomedicine. (2023) 121:155092. doi: 10.1016/j.phymed.2023.155092, PMID: 37804820

[32] Lu S Chen Y An G Tao X Qiao C Chen M . Polyphyllin I exerts anti-hepatocellular carcinoma activity by targeting ZBTB16 to activate the PPARγ/RXRα signaling pathway. Chin Med. (2024) 19:113. doi: 10.1186/s13020-024-00984-0, PMID: 39182119 PMC11344421

[33] Xu F Yang F Qiu Y Wang C Zou Q Wang L . The alleviative effect of C-phycocyanin peptides against TNBS-induced inflammatory bowel disease in zebrafish via the MAPK/Nrf2 signaling pathways. Fish Shellfish Immunol. (2024) 145:109351. doi: 10.1016/j.fsi.2023.109351, PMID: 38171429

[34] Li P Zhu L Song C Wu M Zhu X He S . Triple-functional probiotics with intracellularly synthesized selenium nanoparticles for colitis therapy by regulating the macrophage phenotype and modulating gut microbiota. ACS Nano. (2025) 19:14213–32. doi: 10.1021/acsnano.5c00574, PMID: 40192063

[35] Li J Niu C Ai H Li X Zhang L Lang Y . TSP50 attenuates DSS-induced colitis by regulating TGF-β Signaling mediated maintenance of intestinal mucosal barrier integrity. Adv Sci. (2024) 11:e2305893. doi: 10.1002/advs.202305893, PMID: 38189580 PMC10953580

[36] Goto Y Panea C Nakato G Cebula A Lee C Diez MG . Segmented filamentous bacteria antigens presented by intestinal dendritic cells drive mucosal Th17 cell differentiation. Immunity. (2014) 40:594–607. doi: 10.1016/j.immuni.2014.03.005, PMID: 24684957 PMC4084624

[37] Jiang L Shen Y Guo D Yang D Liu J Fei X . EpCAM-dependent extracellular vesicles from intestinal epithelial cells maintain intestinal tract immune balance. Nat Commun. (2016) 7:13045. doi: 10.1038/ncomms13045, PMID: 27721471 PMC5062543

[38] Yang Y Lv X Zhan L Chen L Tang X Shi Q . Case report: IL-21 and bcl-6 regulate the proliferation and secretion of tfh and tfr cells in the intestinal germinal center of patients with inflammatory bowel disease. Front Pharmacol. (2020) 11:587445. doi: 10.3389/fphar.2020.587445, PMID: 33584264 PMC7873887

[39] Matsuda T . The physiological and pathophysiological role of IL-6/STAT3-mediated signal transduction and STAT3 binding partners in therapeutic applications. Biol Pharm Bull. (2023) 46:364–78. doi: 10.1248/bpb.b22-00887, PMID: 36858565

[40] Oroz J Barrera-Vilarmau S Alfonso C Rivas G Alba ED . ASC pyrin domain self-associates and binds NLRP3 protein using equivalent binding interfaces. J Biol Chem. (2016) 291:19487–501. doi: 10.1074/jbc.M116.741082, PMID: 27432880 PMC5016686

[41] Subramanian N Natarajan K Clatworthy MR Wang Z Germain RN . The adaptor MAVS promotes NLRP3 mitochondrial localization and inflammasome activation. Cell. (2013) 153:348–61. doi: 10.1016/j.cell.2013.02.054, PMID: 23582325 PMC3632354

[42] Pan P Shen M Yu Z Ge W Chen K Tian M . SARS-CoV-2 N protein promotes NLRP3 inflammasome activation to induce hyperinflammation. Nat Commun. (2021) 12:4664. doi: 10.1038/s41467-021-25015-6, PMID: 34341353 PMC8329225

[43] Friedrich M Pohin M Jackson MA Korsunsky I Bullers SJ Rue-Albrecht K . IL-1-driven stromal-neutrophil interactions define a subset of patients with inflammatory bowel disease that does not respond to therapies. Nat Med. (2021) 27:1970–81. doi: 10.1038/s41591-021-01520-5, PMID: 34675383 PMC8604730

[44] Cheng CK Yi M Wang L Huang Y . Role of gasdermin D in inflammatory diseases, from mechanism to therapeutics. Front Immunol. (2024) 15:1456244. doi: 10.3389/fimmu.2024.1456244, PMID: 39253076 PMC11381298

[45] Bulek K Zhao J Liao Y Rana N Corridoni D Antanaviciute A . Epithelial-derived gasdermin D mediates nonlytic IL-1β release during experimental colitis. J Clin Invest. (2020) 130:4218–34. doi: 10.1172/JCI138103, PMID: 32597834 PMC7410065

[46] Khatri V Kalyanasundaram R . Therapeutic implications of inflammasome in inflammatory bowel disease. FASEB J. (2021) 35:e21439. doi: 10.1096/fj.202002622R, PMID: 33774860 PMC8010917

[47] Yin J Sheng B Yang K Sun L Xiao W Yang H . The protective roles of NLRP6 in intestinal epithelial cells. Cell Prolif. (2019) 52:e12555. doi: 10.1111/cpr.12555, PMID: 30515917 PMC6496424

[48] Zou J Yang R Feng R Liu J Wan J . Ginsenoside Rk2, a dehydroprotopanaxadiol saponin, alleviates alcoholic liver disease via regulating NLRP3 and NLRP6 inflammasome signaling pathways in mice. J Pharm Anal. (2023) 13:999–1012. doi: 10.1016/j.jpha.2023.05.005, PMID: 37842661 PMC10568107

[49] Yu J Liu T Guo Q Wang Z Chen Y Dong Y . Disruption of the intestinal mucosal barrier induced by high fructose and restraint stress is regulated by the intestinal microbiota and microbiota metabolites. Microbiol Spectr. (2023) 11:e0469822. doi: 10.1128/spectrum.04698-22, PMID: 36719201 PMC10100858

[50] Park J Son MJ Ho CC Lee SH Kim Y An J . Transcriptional inhibition of STAT1 functions in the nucleus alleviates Th1 and Th17 cell-mediated inflammatory diseases. Front Immunol. (2022) 13:1054472. doi: 10.3389/fimmu.2022.1054472, PMID: 36591260 PMC9800178

[51] Warren CFA Wong-Brown MW Bowden NA . BCL-2 family isoforms in apoptosis and cancer. Cell Death Dis. (2019) 10:177. doi: 10.1038/s41419-019-1407-6, PMID: 30792387 PMC6384907

[52] Cheng E . Molecular control of mitochondrial apoptosis by the BCL-2 family. Blood. (2009) 114:SCI–13. doi: 10.1182/blood.V114.22.SCI-13.SCI-13 PMC2714431 19193868

[53] Czabotar PE Lessene G Strasser A Adams JM . Control of apoptosis by the BCL-2 protein family: implications for physiology and therapy. Nat Rev Mol Cell Biol. (2014) 15:49–63. doi: 10.1038/nrm3722, PMID: 24355989

[54] Nguyen PM Dagley LF Preaudet A Lam N Giam M Fung KY . Loss of Bcl-G, a Bcl-2 family member, augments the development of inflammation-associated colorectal cancer. Cell Death Differ. (2020) 27:742–57. doi: 10.1038/s41418-019-0383-9, PMID: 31296963 PMC7206067

[55] Brown-Suedel AN Bouchier-Hayes L . Caspase-2 substrates: to apoptosis, cell cycle control, and beyond. Front Cell Dev Biol. (2020) 8:610022. doi: 10.3389/fcell.2020.610022, PMID: 33425918 PMC7785872

[56] Li P Zhou L Zhao T Liu X Zhang P Liu Y . Caspase-9: structure, mechanisms and clinical application. Oncotarget. (2017) 8:23996–4008. doi: 10.18632/oncotarget.15098, PMID: 28177918 PMC5410359

[57] Unnisa A Greig NH Kamal MA . Inhibition of caspase 3 and caspase 9 mediated apoptosis: A multimodal therapeutic target in traumatic brain injury. Curr Neuropharmacol. (2023) 21:e270322202653. doi: 10.2174/1570159X20666220327222921, PMID: 35339178 PMC10227914

[58] Zeng M Wang K Wu Q Ding J Xie D Qi X . Dissecting caspase-2-mediated cell death: from intrinsic PIDDosome activation to chemical modulation. Protein Cell. (2024) 15:889–905. doi: 10.1093/procel/pwae020, PMID: 38676703 PMC11637483

[59] Wang K Yin J Chen J Ma J Si H Xia D . Inhibition of inflammation by berberine: Molecular mechanism and network pharmacology analysis. Phytomedicine. (2024) 128:155258. doi: 10.1016/j.phymed.2023.155258, PMID: 38522318

[60] Chao T Shih HT Hsu SC Chen PJ Fan YS Jeng YM . Autophagy restricts mitochondrial DNA damage-induced release of ENDOG (endonuclease G) to regulate genome stability. Autophagy. (2021) 17:3444–60. doi: 10.1080/15548627.2021.1874209, PMID: 33465003 PMC8632313

[61] Zaidi D Wine E . Regulation of nuclear factor kappa-light-chain-enhancer of activated B cells (NF-κβ) in inflammatory bowel diseases. Front Pediatr. (2018) 6:317. doi: 10.3389/fped.2018.00317, PMID: 30425977 PMC6218406

[62] Chawla M Mukherjee T Deka A Chatterjee B Sarkar UA Singh AK . An epithelial Nfkb2 pathway exacerbates intestinal inflammation by supplementing latent RelA dimers to the canonical NF-κB module. Proc Natl Acad Sci U.S.A. (2021) 118:e2024828118. doi: 10.1073/pnas.2024828118, PMID: 34155144 PMC8237674

[63] He Y Sun MM Zhang GG Yang J Chen KS Xu WW . Targeting PI3K/Akt signal transduction for cancer therapy. Signal Transduct Target Ther. (2021) 6:425. doi: 10.1038/s41392-021-00828-5, PMID: 34916492 PMC8677728

[64] Lan M Lin C Zeng L Hu S Shi Y Zhao Y . Linderanine C regulates macrophage polarization by inhibiting the MAPK signaling pathway against ulcerative colitis. BioMed Pharmacother. (2024) 178:117239. doi: 10.1016/j.biopha.2024.117239, PMID: 39098180

[65] Stankey CT Bourges C Haag LM Turner-Stokes T Piedade AP Palmer-Jones C . A disease-associated gene desert directs macrophage inflammation through ETS2. Nature. (2024) 630:447–56. doi: 10.1038/s41586-024-07501-1, PMID: 38839969 PMC11168933

[66] Rajput A Ware CF . Tumor necrosis factor signaling pathways. Encyclopedia Cell Biol. (2016) 3:354–63. doi: 10.1016/b978-0-12-394447-4.30048-7

[67] Kong C Yang M Yue N Zhang Y Tian C Wei D . Restore intestinal barrier integrity: an approach for inflammatory bowel disease therapy. J Inflammation Res. (2024) 17:5389–413. doi: 10.2147/JIR.S470520, PMID: 39161679 PMC11330754

[68] Calafiore M Fu YY Vinci P Arnhold V Chang WY Jansen SA . A tissue-intrinsic IL-33/EGF circuit promotes epithelial regeneration after intestinal injury. Nat Commun. (2023) 14:5411. doi: 10.1038/s41467-023-40993-5, PMID: 37669929 PMC10480426

[69] Ma T Gan G Cheng J Shen Z Zhang G Liu S . Engineered probiotics enable targeted gut delivery of dual gasotransmitters for inflammatory bowel disease therapy. Angew Chem Int Ed Engl. (2025) 64:e202502588. doi: 10.1002/anie.202502588, PMID: 40091878

[70] Wee P Wang Z . Epidermal growth factor receptor cell proliferation signaling pathways. Cancers. (2017) 9:52. doi: 10.3390/cancers9050052, PMID: 28513565 PMC5447962

[71] Sabbah DA Hajjo R Sweidan K . Review on epidermal growth factor receptor (EGFR) structure, signaling pathways, interactions, and recent updates of EGFR inhibitors. Curr Top Med Chem. (2020) 20:815–34. doi: 10.2174/1568026620666200303123102, PMID: 32124699

[72] Stakenborg M Verstockt B Meroni E Goverse G De Simone V Verstockt S . Neutrophilic HGF-MET signalling exacerbates intestinal inflammation. J Crohns Colitis. (2020) 14:1748–58. doi: 10.1093/ecco-jcc/jjaa121, PMID: 32556102

[73] Failla CM Carbone ML Ramondino C Bruni E Orecchia A . Vascular endothelial growth factor (VEGF) family and the immune system: activators or inhibitors? Biomedicines. (2025) 13:6. doi: 10.3390/biomedicines13010006, PMID: 39857591 PMC11763294

[74] Di Mola FF Friess H Zhu ZW Koliopanos A Bley T Sebastiano PD . Nerve growth factor and Trk high affinity receptor (TrkA) gene expression in inflammatory bowel disease. Gut. (2000) 46:670–9. doi: 10.1136/gut.46.5.670, PMID: 10764711 PMC1727937

[75] Dothel G Barbaro MR Boudin H Vasina V Cremon C Gargano L . Nerve fiber outgrowth is increased in the intestinal mucosa of patients with irritable bowel syndrome. Gastroenterology. (2015) 148:1002–11.e4. doi: 10.1053/j.gastro.2015.01.042, PMID: 25655556

