# Supplementary material for: Evaluation of the safety and efficacy of Sophorae Flavescentis Radix extract in the treatment of inflammatory bowel disease based on zebrafish models
Source: Front Immunol. 2025 Nov 27;16:1722777. doi: 10.3389/fimmu.2025.1722777 (PMC12696176; doi:10.3389/fimmu.2025.1722777)
Supplement: Supplementary file 1 [file Table1.docx]

Supplementary Material

# Supplementary Tables

Supplementary Table 1 Primer sequences

| Gene | Forward Primer sequence（5'-3'） | Reverse Primer sequence（5'-3'） |
| --- | --- | --- |
| FoxO4 | GCGATTCGAATGTGACGTGG | TACACTGTGAGGCACGAACC |
| IL-6 | TCCTCAAACCTTCAGACCGC | CGTTCACCAGGACTGAGGTC |
| TGFB | TGGAAAGCAACTGCAATGGC | ACTCTGCCTCCAGATAGCCA |
| BCL-6 | AAATGTGACCGTTGCCAAGC | CGGTTTCTCTCCGGTGTGAA |
| NLRP3 | GCACACATGTTTCACACGCT | AGAGAGGCTGTGTGAAAGGC |
| NLRP6 | CAGACTGTTTGGTGCTGTGC | CCCCGATCTCTGACAGATGC |
| GSDMD | CTGAAAGACGCTGCTCTGGA | TCCTTGAGCAGCATCACGTT |
| IL-1β | CTGAAATGATGGCATGCGGG | GCTCATTGCAAGCGGATCTG |
| STAT1 | TTTTCGTGACTCCTCCACCG | GATCCGATGCCGCTTTAGGA |
| BAD | TGCTGGAAACTGGAGTTGCA | CACAAAGCAGGAGGAGCTGA |
| BID | AGCTTGTGGAGATGGCCTTC | AAAACTGCACCACTGTCCCA |
| ENDOG | CGGAAGCTCGGACAGAAAGT | CCCTGTCAAATCCACTCCCC |
| BCL2 | GCGGAGGGAACAACTCTGAA | ATCCCGTAACACCCGGTAGA |
| CASP2 | CAGCTGTACTTCCCTGCACA | ATCCACCTCTCCTCCCCTTC |
| CASP9 | CGCCCTCCTGTCATTTCTGT | GCAGGCTTTCAGGTCTCAGT |
| RELA | CGGCATCCTCCTCAGACATC | TATTCTCTCCCCTGGCTGCT |
| PIK3CB | GGAGGCGCAGACATATCCTC | AGAACAGGCAGGAATGGTCG |
| CAPN1 | AATGGGTGGACGTGGTGATC | TCGTAACAGCCGTTCAGCTT |
| AKT1 | ACGATCACCATCACACCACC | CGCTGGCCGAGTATGAGAAT |
| BRAF | CCACTCCACAGCAGCTACAA | GTAGAGACTCGAGCCTTCGC |
| TNF | CTCGCTGCAGTTGCTTTTGT | TTTCCGTGGTCTGAGGAAGC |
| MAPK8 | GTTCACCGTTTTGAAGCGCT | CCCGTTTGGCATGAGTTTGG |
| EGF | GCGTCTGTACTGGTGTGACA | GACCCAGAGCACGTTCTCAA |
| EGFR | GACGACCGCATGCATTTACC | TTCAGGCTCACAGAGTGCAG |
| MET | GCCGGAGACTGTTACAACCA | GAGGATGTAGTGCTCTCCGC |
| KDR | GGTGGTCATTCCCTGTCTGG | AGATGAAGCCCCTCCTGCTA |
| NTRK1 | CTGCCCAAAAGAAGTGCACC | CGAGGCGACTGTAGATGTCC |
| β-actin | ATGGATGAGGAAATCGCTGCC | CTCCCTGATGTCTGGGTCGTC |

Supplementary Table 2 Molecular docking

| Gene | Binding Affinity (kcal/mol) | | | | | | | | |
| --- | --- | --- | --- | --- | --- | --- | --- | --- | --- |
|  | Matrine | Oxymatrine | | | Sophoridine | | Oxysophoridine | | Kurarinone |
| AKT1 | -7.3 | | -7.5 | -7.4 | | -7.6 | | -8.2 | |
| BCL2 | -8.1 | | -8.1 | -7.9 | | -7.8 | | -8.2 | |
| BRAF | -7.6 | | -8.1 | -8.0 | | -8.0 | | -8.3 | |
| CAPN1 | -6.2 | | -6.4 | -5.9 | | -6.7 | | -6.7 | |
| EGFR | -7.6 | | -10.4 | -8.3 | | -7.8 | | -8.9 | |
| KDR | -6.8 | | -7.4 | -6.4 | | -6.8 | | -7.4 | |
| MET | -6.6 | | -6.8 | -6.7 | | -7.0 | | -7.7 | |
| TNF | -7.6 | | -6.9 | -6.8 | | -6.8 | | -8.1 | |
| STAT1 | -6.2 | | -6.6 | -6.7 | | -6.3 | | -6.6 | |
| RELA | -7.4 | | -6.6 | -6.7 | | -7.3 | | -7.1 | |

Supplementary Table 3 PDB IDs

| Protein | PDB ID |
| --- | --- |
| AKT1 | 7WM2 |
| EGFR | 2XKN |
| BCL2 | 4HW3 |
| BRAF | 3OG7 |
| TNF | 4QQL |
| STAT1 | 3VNF |
| KDR | 2R1Y |
| MET | 1MJQ |
| NTRK1 | 4GT5 |
| CAPN1 | 8GX3 |

# Supplementary Figures

**HPLC Fingerprint:**

Chromatographic conditions: With octadecylsilane-bonded silica gel as the filler. Using acetonitrile -[0.01mol/L ammonium acetate solution (pH adjusted to 8.1 with concentrated ammonia test solution)] (3:2) as the mobile phase A and 0.01mol/L ammonium acetate solution (pH adjusted to 8.1 with concentrated ammonia test solution) as the mobile phase B.

Identified markers: **Matrine** (Rt=36.310 min).


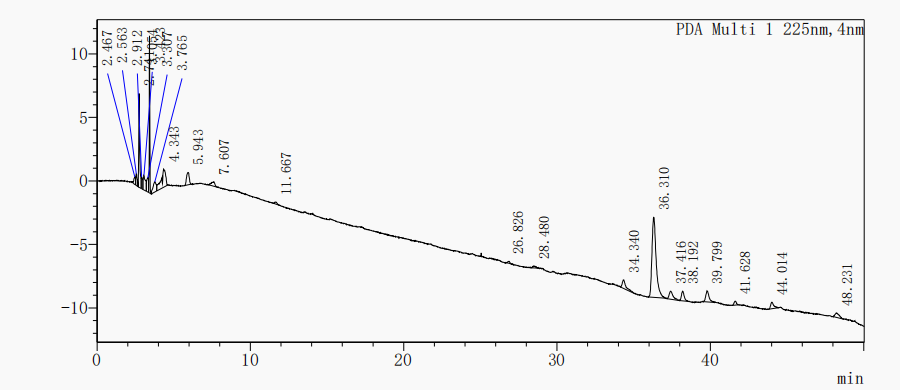


Fig. 1 HPLC diagram of matrine

Identified markers: **Oxymatrine** (Rt=15.788 min)


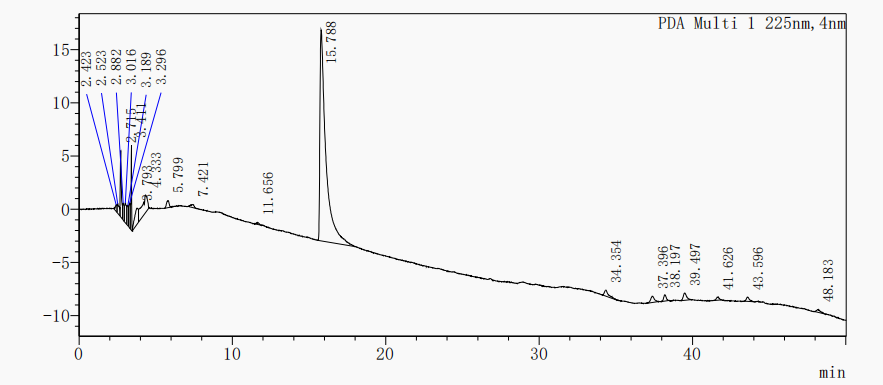


Fig. 2 HPLC diagram of Oxymatrine

**Kushen** extract: **Matrine** (Rt=36.530 min), **Oxymatrine** (Rt=15.588 min)


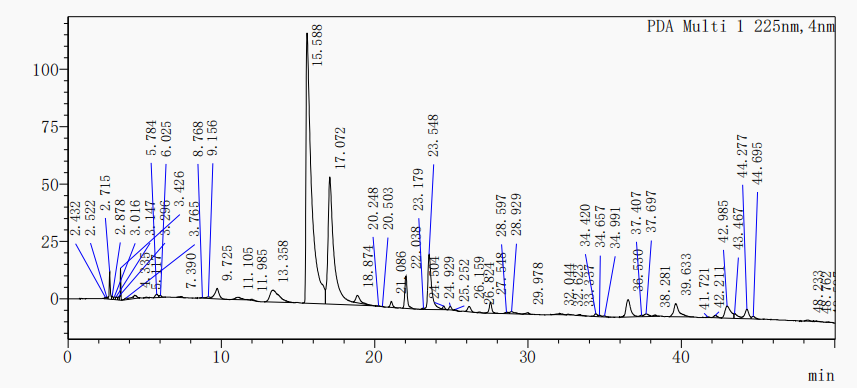


Fig. 3 HPLC diagram of Kushen extract
